# NCAM Regulates Inhibition and Excitability in Layer 2/3 Pyramidal Cells of Anterior Cingulate Cortex

**DOI:** 10.3389/fncir.2017.00019

**Published:** 2017-03-23

**Authors:** Xuying Zhang, Chelsea S. Sullivan, Megan B. Kratz, Michael R. Kasten, Patricia F. Maness, Paul B. Manis

**Affiliations:** ^1^Department of Biochemistry and Biophysics, The University of North Carolina at Chapel HillChapel Hill, NC, USA; ^2^Department of Otolaryngology/Head and Neck Surgery, The University of North Carolina at Chapel HillChapel Hill, NC, USA; ^3^Department of Cell Biology and Physiology, The University of North Carolina at Chapel HillChapel Hill, NC, USA

**Keywords:** mouse, channelrhodopsin, brain slice, patch clamp, laser scanning photostimulation

## Abstract

The neural cell adhesion molecule (NCAM), has been shown to be an obligate regulator of synaptic stability and pruning during critical periods of cortical maturation. However, the functional consequences of NCAM deletion on the organization of inhibitory circuits in cortex are not known. In vesicular gamma-amino butyric acid (GABA) transporter (VGAT)-channelrhodopsin2 (ChR2)-enhanced yellow fluorescent protein (EYFP) transgenic mice, NCAM is expressed postnatally at perisomatic synaptic puncta of EYFP-labeled parvalbumin, somatostatin and calretinin-positive interneurons, and in the neuropil in the anterior cingulate cortex (ACC). To investigate how NCAM deletion affects the spatial organization of inhibitory inputs to pyramidal cells, we used laser scanning photostimulation in brain slices of VGAT-ChR2-EYFP transgenic mice crossed to either NCAM-null or wild type (WT) mice. Laser scanning photostimulation revealed that NCAM deletion increased the strength of close-in inhibitory connections to layer 2/3 pyramidal cells of the ACC. In addition, in NCAM-null mice, the intrinsic excitability of pyramidal cells increased, whereas the intrinsic excitability of GABAergic interneurons did not change. The increase in inhibitory tone onto pyramidal cells, and the increased pyramidal cell excitability in NCAM-null mice will alter the delicate coordination of excitation and inhibition (E/I coordination) in the ACC, and may be a factor contributing to circuit dysfunction in diseases such as schizophrenia and bipolar disorder, in which NCAM has been implicated.

## Introduction

Efficient transmission of information in the brain requires an intricate coordination of excitation and inhibition (E/I coordination; Mullins et al., 2016), which is established during development and can be modified later by experience (Bartley et al., 2008; Gibson et al., 2008). Gamma-amino butyric acid (GABA) GABAergic interneurons are critical determinants of cortical network function. Alteration in GABAergic inhibition disrupts the E/I coordination and is associated with cognitive deficits in neurodevelopmental disorders, such as schizophrenia and autism (Chu and Anderson, 2015; Gonzalez-Burgos et al., 2015; Nelson and Valakh, 2015). While many advances have been made in understanding how excitatory synapses are generated and refined (Caroni et al., 2014), the cellular and molecular mechanisms regulating inhibitory synapse formation and elimination are incompletely understood. Basket cells are a major subtype of GABAergic interneuron whose profuse axonal arbors form synapses on the somatic and proximal dendritic domains of pyramidal neurons (Freund and Katona, 2007; Fino and Yuste, 2011), and express parvalbumin. Basket interneurons coordinately inhibit and synchronize output pyramidal cell groups (Klausberger et al., 2004; Sohal et al., 2009), which is thought to be important for cognitive functions in the prefrontal cortex (Bartos et al., 2007; Lewis et al., 2012). Other inhibitory interneurons in the upper layers of cortex include somatostatin-positive cells (~30%; a subset of which are calretinin expressing) and 5HT3aR-expressing cells (30%; a subset of which are also calretinin expressing; Xu et al., 2010; Rudy et al., 2011). Cells expressing somatostatin are thought to be involved in a modulatory role aiding in habituation (Kato et al., 2015) or desynchronization of parvalbumin-expressing (basket and chandelier) neurons (Chen et al., 2015). Despite the central role of these various interneurons in cortical networks, the molecular mechanisms regulating the formation of prefrontal cortical inhibitory circuits are poorly understood.

Recently, we identified a role for the neural cell adhesion molecule (NCAM) in elimination of excess perisomatic inhibitory synapses in the developing anterior cingulate cortex (ACC) by the action of ephrinA5 repellent ligands and EphA3 receptors (Brennaman et al., 2013). Genetic deletion of NCAM, EphA3 or ephrinA2/A3/A5 in null mutant mice increased the number and size of GABAergic perisomatic synaptic puncta, accompanied by increased amplitudes and faster kinetics of miniature inhibitory postsynaptic currents (IPSCs) in the NCAM-null ACC (Brennaman et al., 2013). Furthermore, ephrinA5 treatment of ACC slice preparations promoted the loss of GABAergic perisomatic inputs (Brennaman et al., 2013). Consistent with a possible altered cortical E/I connectivity, NCAM deficient mice exhibit alterations in aggression, anxiety, fear conditioning and social motivation, which are all behaviors that may be influenced or modulated by prefrontal networks (Stork et al., 1999; Senkov et al., 2006; Calandreau et al., 2010; Kochlamazashvili et al., 2010). Genetic polymorphisms or dysregulation of NCAM (Arai et al., 2004; Atz et al., 2007; Anney et al., 2010; Gray et al., 2010; Varea et al., 2012) and ephrinA/EphA (Wilson et al., 2006; Ikeda et al., 2010; Ayalew et al., 2012; Casey et al., 2012) have been linked to schizophrenia, autism and bipolar disorder.

Neurodevelopmental dysregulation of inhibitory synapses could potentially disrupt E/I coordination in cortical networks, and may effect homeostatic changes in the strength of other synaptic inputs or in the intrinsic excitability of cells. To investigate how mechanisms that control inhibitory synapse remodeling in development affect the overall functional organization of ACC networks and to evaluate the consequences of disrupting NCAM function, we used an optogenetic approach in which channelrhodopsin-2 (ChR2) fused to enhanced yellow fluorescent protein (EYFP) was expressed in GABAergic interneurons under control of the vesicular GABA transporter (VGAT) in VGAT-ChR2-EYFP mice (Zhao et al., 2011). We used laser-scanning photostimulation to activate VGAT-expressing interneurons (Wang et al., 2007; Kätzel et al., 2011; Yizhar et al., 2011; Zhao et al., 2011) in the ACC of NCAM-null and wild type (WT) mice, while recording from target layer 2/3 pyramidal cells. We found that layer 2/3 pyramidal cells in NCAM-null mice received greater inhibitory input from nearby cells. We also found that pyramidal cell excitability was increased in NCAM-null mice. Such alterations in inhibitory strength and intrinsic excitability resulting from deficits in NCAM-dependent synapse remodeling during postnatal development could contribute to functional deficits affecting behaviors regulated by prefrontal cortical networks.

## Materials and Methods

### Mice

Bacterial artificial chromosome (BAC) transgenic VGAT-hChR2 (H134R)-EYFP mice (line 8; Zhao et al., 2011) were crossed with NCAM-null mutant mice (C57Bl/6 background) to obtain experimental mice that were NCAM-null (Cremer et al., 1994; Hata et al., 2007), and heterozygous for VGAT-hChR2-EYFP. Control data were taken from heterozygous VGAT-hChR2-EYFP mice crossed to WT C57Bl/6 mice. Heterozygous NCAM+/− mice have been reported to exhibit behavioral and biochemical phenotypes that are intermediate between WT and NCAM-null mice (Jurgenson et al., 2012), and therefore we did not use them in these experiments. VGAT-ChR2-EYFP mice exhibit 93% co-localization of EYFP and GAD67 in the cerebral cortex indicating that the vast majority of ChR2-EYFP expressing cells are inhibitory neurons (Zhao et al., 2011). VGAT-hChR2-EYFP mice used in all experiments except those testing the effects of tetrodotoxin (TTX) were obtained directly from the originating laboratory (Dr. Gouping Feng). Experiments testing the effects of TTX used the same line of mice, but obtained from Jackson Laboratories (stock #14548). This study was carried out in accordance with the recommendations of University of North Carolina at Chapel Hill Institutional Animal Care and Use Committee and Division of Laboratory Animal Medicine, in accordance with NIH and USDA guidelines. All protocols were approved by the North Carolina at Chapel Hill Institutional Animal Care and Use Committee.

### Immunofluorescence Labeling of Interneuron Subtypes

P21 and P40 VGAT-ChR2-EYFP mice were anesthetized, perfused transcardially with 4% paraformaldehyde, and processed for staining as described (Demyanenko et al., 1999). Brains were removed and postfixed in 4% paraformaldehyde overnight at 4°C, cryoprotected in a 10%–30% sucrose series, and cryosectioned (16 μm). Sections were permeabilized and blocked in 0.5% Triton in phosphate buffered saline with 10% normal horse serum for 1 h at room temperature. Sections were incubated with antibodies against NCAM intracellular domain (ICD; OB11, Sigma-Aldrich, 1:200; Liu and Martin, 2006), NCAM extracellular domain (ECD; Ab5032, Millipore, 1:200; Le Pichon and Firestein, 2008), PSA (5A5, gift of Urs Rutishauser, 1:1000; Wang et al., 2006), VGAT (131-003, Synaptic Systems, 1:500; Stensrud et al., 2013), parvalbumin (Parv-19, Sigma-Aldrich, 1:500; Cerkevich et al., 2013), calretinin (37C9, Synaptic Systems, 1:200; Toader et al., 2013), somatostatin (Ab354, Chemicon, 1:200; Xu et al., 2008), or EYFP (ab13970, Abcam, 1:500; Hunter et al., 2011) overnight at 4°C in 1% normal horse serum in phosphate buffered saline, then with AlexaFluor-488-labeled, AlexaFluor-555–labeled, or AlexaFluor-647 secondary antibodies (all at 1:500) for 1 h at room temperature. With the exception of the calretinin antibody, all primary antibodies used can be found in the Journal of Comparative Neurology antibody database. The calretinin antibody is in the Antibody Registry database (RRID:AB_2619904)^1^. Nuclei were stained with Hoechst (Thermo-Fisher), and sections were mounted in ProLong Gold Antifade (Life Technologies). Images were captured on an Olympus LSM710 confocal microscope using a ×60 objective with ×2 optical zoom. Cryosections from P21 VGAT-ChR2-EYFP mice were co-stained for EYFP and markers of interneuron types (parvalbumin, calretinin and somatostatin). The percentage of EYFP positive neurons expressing each marker was determined by scoring cells under widefield fluorescence microscopy (Zeiss Axioplan 2 microscope, 40× oil immersion) within nine coronal cryosections per marker from three mice in layer 2/3 of the ACC. Perisomatic puncta were defined previously (Brennaman et al., 2013) as labeled puncta that were within 2 μm of the Hoechst-labeled nucleus. At least 600 EYFP positive cells were analyzed per mouse for each interneuron marker.

### Preparation and Incubation of Slices for Electrophysiology

Mice (P30–P40) were anesthetized with ketamine (80 mg/kg)-xylazine (8 mg/kg), prior to decapitation. Coronal slices (350 μm) of the ACC were prepared with a vibrating tissue slicer (Leica 1000S, Germany) in ice-cold oxygenated low-sodium artificial cerebrospinal fluid (ACSF) containing (in mM): 135 N-methyl-D-glucamine, 20 choline chloride, 20 NaHCO_3_, 2.2 KCl, 0.5 CaCl_2_, 1.5 MgSO_4_, 1.2 NaH_2_PO_4_, 10 D-glucose. The pH was adjusted to 7.3–7.4 with HCl, and the solution was gassed with 95% O_2_/5% CO_2_. Prior to recording, the slices were incubated for 30 min at 34°C, and then maintained at room temperature for at least 1 h in ACSF. ACSF contained (in mM): 125 NaCl, 2.5 KCl, 2 CaCl_2_, 1.3 MgSO_4_, 20 D-glucose, 1.25 NaH_2_PO_4_, 26 NaHCO_3_, and was saturated with 95% O_2_/5% CO_2_. The normal ACSF solution was supplemented with 10 μM 6-cyano-7-nitroquinoxaline-2,3-dione (CNQX) and 50 μM D-2-amino-5-phosphonovalerate (D-APV) to block excitatory transmission during mapping. In some experiments, 500 μM kynurenic acid was used instead of CNQX and D-APV. ACSF and pipette solution components were obtained from Sigma-Aldrich (St. Louis, MO, USA). D-APV, CNQX and TTX was obtained from Tocris/Biotechne (Minneapolis, MN, USA). TTX was stored as aliquots in 1 mM acetic acid at −20°C, and diluted on the day of use to 1 μM. TTX was handled in accordance with a protocol approved by the UNC department of Environment and Health Science, and was inactivated in a trap containing sodium hypochlorite after use. AlexaFluor dyes (488, 532, 568) and Lucifer Yellow were purchased from Molecular Probes (Eugene, OR, USA) and Invitrogen (Carlsbad, CA, USA).

### Photostimulation Mapping in 2/3 Layer of ACC

Slices were placed in a fast-flow recording chamber on an upright epifluorescence microscope (Axioskop FS2, Zeiss, Germany). To identify ChR2-expressing neurons, the fluorescence of EYFP fused to ChR2 was detected with a Retiga-2000DC or R1 camera (QImaging, Surrey, BC, Canada) with a 40× or 63× water immersion objective. Excitation was provided by a 505 nm light emitting diode (LED), and fluorescence separated from excitation light using a Semrock EYFP-2427B filter set and dichroic mirror. Transmission of blue light that could excite ChR2 was minimal through this filter set, and thus avoided stimulating cells when examining fluorescence. The 505 nm LED was turned off and removed from the optical pathway prior to performing mapping.

Patch pipettes were pulled from 1.2 mm borosilicate glass (Sutter Instruments) with a Sutter P-2000 puller. For current clamp recordings, electrodes (6–8 MΩ) were filled with a potassium-based internal solution, which contained (in mM): 126 K-gluconate, 6 KCl, 2 NaCl, 10 HEPES, 0.2 EGTA, 4 ATP-Mg, 0.3 GTP-Na and 10 phosphocreatine-Tris. The pH was adjusted to 7.2 with KOH and the osmolarity set to 280–295 mOsm (the osmolarity was adjusted with sucrose if needed). The intrinsic excitability of interneurons was measured with step current pulses, 0.5–1 s in duration, injected through the recording electrode in current clamp mode. For voltage clamp recordings, the recording pipette contained (in mM) 68 CsMetSO_3_, 72 CsCl, 5 EGTA, 10 HEPES, 4 ATP-Mg, 0.3 GTP-Na, 10 creatine phosphate and 3 QX–314 chloride, pH 7.2. The high chloride concentration in the pipette set *E*_Cl_ to ~−14 mV, so that IPSCs had a large driving force for cells voltage-clamped at −60 mV, which facilitated their detection. Except for Figure 10, voltage and current-clamp recordings were made with an Axopatch 200A amplifier (Axon Instruments/Molecular Devices). The I-fast mode of the amplifier was used for current clamp recordings to minimize distortion of action potential shape. In Figure 10, current-clamp recordings were made with a Multiclamp 700B amplifier (Axon Instruments/Molecular Devices). All current and voltage measurements used for mapping were digitized at 200–400 kHz with a NI6052E National Instruments multifunction data acquisition card, and downsampled to 10 kHz. No compensation was used in voltage-clamp, and membrane potentials are presented without correction for the liquid junction potential between pipette and bath solutions (typically −11 mV for the experiments using K-gluconate, and −4 mV for the Cs-based solution used for voltage-clamp). Experiments were controlled and data acquired with the program ACQ4 (Campagnola et al., 2014), available at www.acq4.org. For consistency, all measurements of intrinsic excitability and mapping were made at room temperature (22–25°C), with the exception of experiments measuring pyramidal cell excitability shown in Figure 10, which were made at 34°C.

The recording electrodes in mapping experiments also contained 50–100 μM AlexaFluor 568 for morphological identification of cells. AlexaFluor 568 fluorescence was excited with a 530 nm LED through the epi-illumination train, and the fluorescence was separated from the excitation light with a TRITC filter (Zeiss) set. Recording electrodes in the series of cells used to examine the effects of TTX contained AlexaFluor 532 or 488, whereas those used in the experiments examining intrinsic excitability at 34°C used a sub-saturating concentration of Lucifer Yellow (K^+^ salt); appropriate TRITC and FITC filter sets (Zeiss) or Lucifer Yellow (Chroma) were used to examine fluorescent cells. Cells were imaged with the CCD camera at the end of the recording session. In some cases, the slices were fixed and re-examined on a confocal or custom multiphoton microscope.

**Figure 1:**
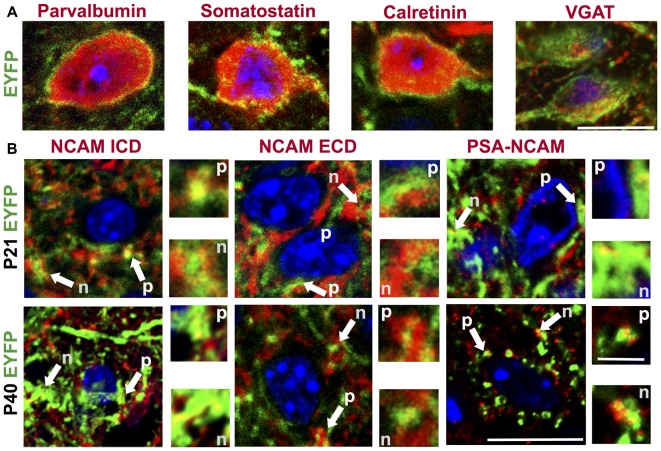
**Characterization of interneuron populations and neural cell adhesion molecule (NCAM) expression in the anterior cingulate cortex (ACC) of vesicular gamma-amino butyric acid (GABA) transporter (VGAT)-channelrhodopsin-2 (ChR2)-enhanced yellow fluorescent protein (EYFP) mice. (A)** Representative confocal images of co-immunostaining of EYFP (green) and the interneuron markers parvalbumin, somatostatin, calretinin and VGAT (each shown in red). Nuclei were stained with Hoechst (blue). Scale bar is 10 μm. **(B)** Representative images of ACC layer 2/3 stained with antibodies to the NCAM intracellular domain (ICD), NCAM extracellular domain (ECD) and PSA-NCAM (red) co-labeled with EYFP (green) and Hoechst (blue). Representative images from age P21 and P40 are shown, along with magnified areas demonstrating colocalization. Arrows indicate perisomatic puncta (p) or neuropil (n) and are shown in higher magnification in outset boxes. Scale bars are 10 μm in the full images and 2.5 μm in the outset boxes.

**Figure 2:**
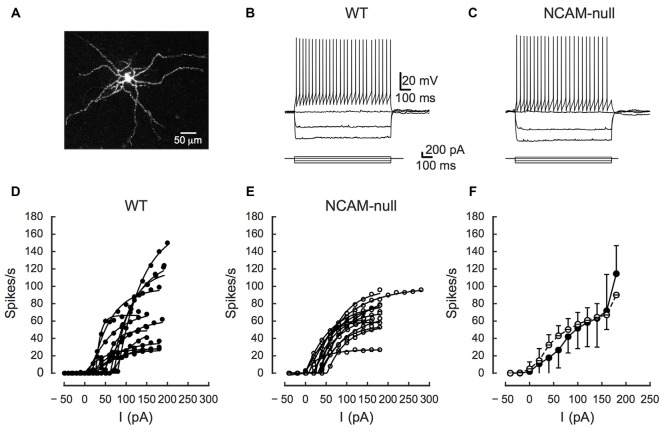
**Comparisons of firing rates of interneurons between wild type (WT) and NCAM-null mice in ACC. (A)** Morphology of a ChR2-expressing cell layer 2/3. The cell was stained with AlexaFluor 568 while recording. **(B)** Interneuron from a WT mouse showing rapid and non-adapting firing (upper traces) in response to intrasomatic current injections (lower traces). **(C)** Interneuron from an NCAM-null mouse showing rapid and non-adapting firing (upper traces) in response to intrasomatic current injections (lower traces). **(D)** Firing frequency as a function of injected current (frequency-current [FI] curves) for 13 interneurons from WT mice. Points are measured firing rates at each current level and the lines are fits for each cell’s FI curve to Equation 1. **(E)** FI curves for 13 interneurons from NCAM-null mice, plotted in the same manner as in panel **(D)**. **(F)** Summary showing mean and standard deviation (SD) of FI curves for cells from WT (solid line and filled symbols) and NCAM-null (dashed line and open symbols) mice (for details, see Table 1). Calibration bars in **(B)** apply to **(C)**.

**Figure 3:**
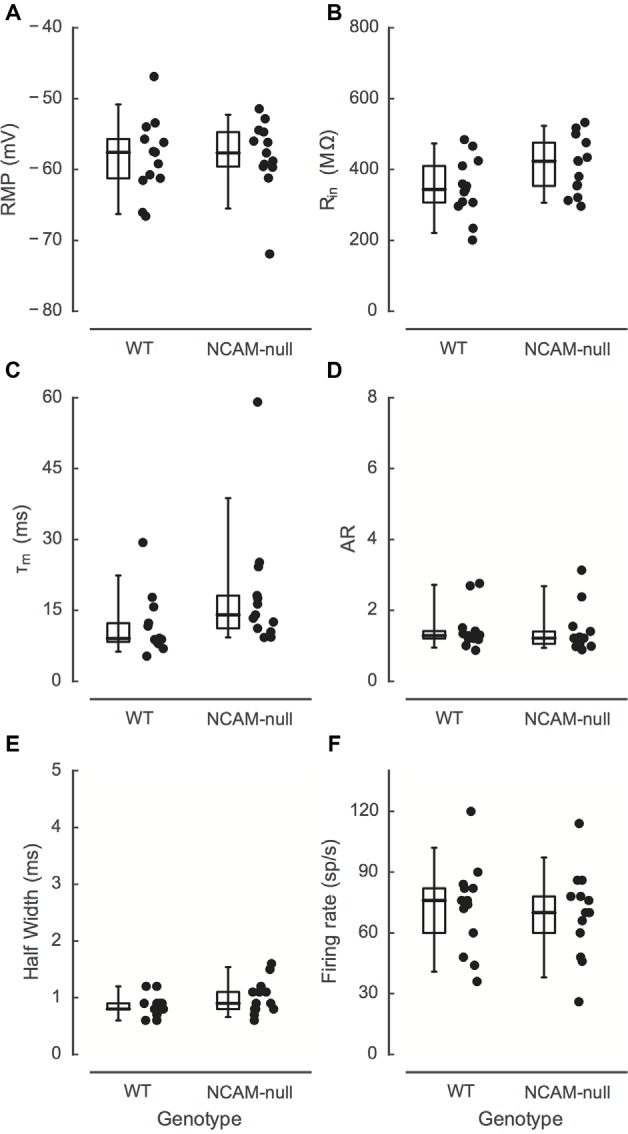
**Comparison of intrinsic electrophysiologic measures between WT and NCAM-null mice. (A)** Resting membrane potential. **(B)** Input resistance. **(C)** Membrane time constant. **(D)** Adaptation ratio. **(E)** Action potential width at half-height. **(F)** Maximal firing rate, determined from equation 1. None of these parameters are different between the genotypes. Solid symbols: data from WT mice; open symbols: data from NCAM-null mice. Boxes indicate median, interquartile (25, 75%) values, and 5%–95% values. For measurement details, see “Materials and Methods” Section.

**Figure 4:**
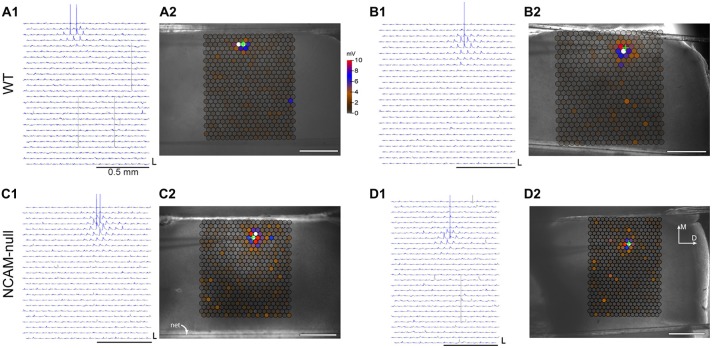
**Laser scanning photostimulation maps for interneurons in WT and NCAM-null mice. (A1)** Shows traces and evoked responses for all locations tested for a WT cell, in response to light flashes at 40 ms. The traces are colored in gray, except for a window from 40 to 60 ms, which is colored in blue to help distinguish evoked from spontaneous depolarizations and spikes. Spikes in the upper rows of each set of traces are cropped. Panel **(A2)** shows a map of maximal amplitudes at each location superimposed on images of the slice, with sites eliciting action potentials colored in white. The recording electrode is visible to the left of the maps in **(A2,B2,C2,D2)**; the green cross indicates the position of the recorded cell in each panel. The midline of the brain is towards the top, and the dorsal edge is to the right (indicated in panel **D2**). The long horizontal strips are the nylon strands holding the slice in the chamber. The scale bar between panels **(A2,B1)** applies to all maps. At the power level used for mapping, most cells generated an action potential only when the light was flashed on or near the cell body, in one or two locations. **(B1,B2)** Evoked responses and amplitude map for another WT cell, as in **(A)**. Panels **(C1,C2,D1,D2)** show responses of two cells from NCAM-null mice mapped at the same power levels as in **(A,B)**, in the same format. The calibration bar in **(A1,B1,C1,D1)** is 10 mV × 100 ms. The scale bars below the maps in **(A1,B1,C1,D1)**, and inset on the images in **(A2,B2,C2,D2)** are 0.5 mm.

**Figure 5:**
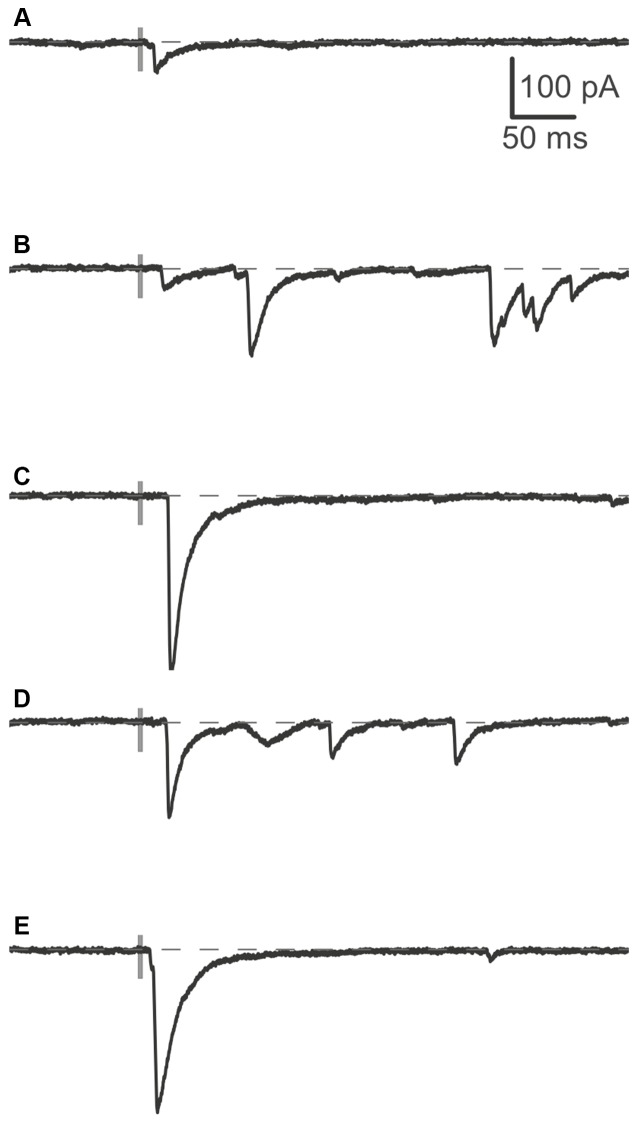
**Responses of a pyramidal cell to photostimulation. (A–E)** Individual traces showing inhibitory postsynaptic currents (IPSCs) evoked by light stimulation at different sites in a map (maps are shown in detail in Figure 6). The timing of the light stimulation is shown by the gray bar on the trace. At some sites, the stimulation resulted in multiple evoked responses in succession **(B,D)**. Recordings from WT pyramidal cell.

**Figure 6:**
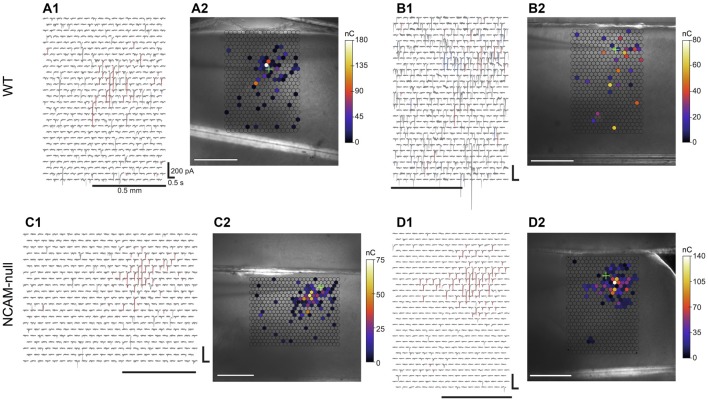
**Laser scanning photostimulation maps for pyramidal cells in WT and NCAM-null mice in voltage-clamp. (A1)** Traces recorded at each site in the map for one example pyramidal cell from a WT mouse. Sites at which the *Z* score was >2.575 are colored in red for the time window 10–40 ms after the light flash. **(A2)** Color map of the charge associated with the first evoked event. Only sites where the *Z* score was >2.575 are colored; other sites are transparent. The color scale is shown to the right. The location of the cell body is marked by a green cross. **(B1,B2)** Example from another pyramidal cell from at WT mouse, shown in the same format as in **(A1,A2)**. This cell had a higher frequency of spontaneous IPSCs. **(C1,C2)** Example from a cell from an NCAM-null mouse, in the same format as **(A1,A2)**. **(D1,D2)** Example traces and map from another cell from an NCAM–null mouse. The calibration bars for the traces in **(A1,B1,C1,D1)** are 200 pA × 0.5 s. The scale bars below the maps in **(A1,B1,C1,D1)**, and inset on the images in **(A2,B2,C2,D2)** are 0.5 mm.

**Figure 7:**
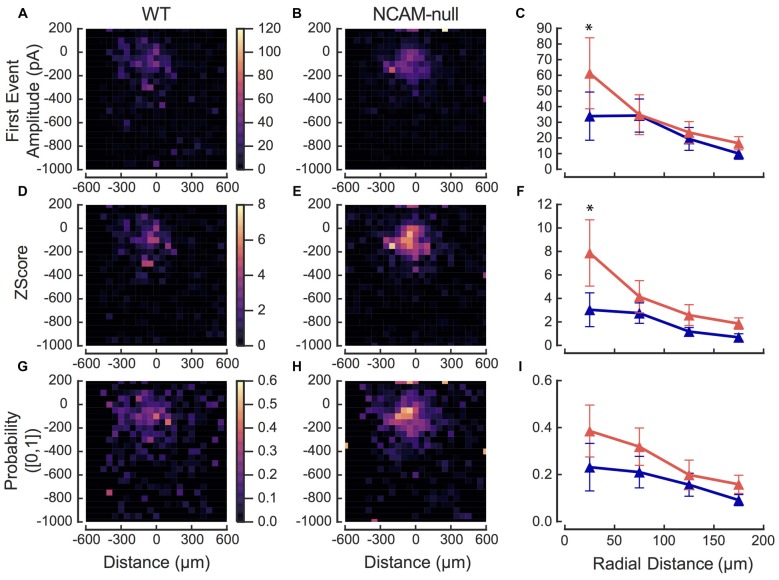
**Summary maps of photostimulation sites that produce inhibitory currents in pyramidal cells**. Summary maps for each genotype were generated by first averaging repeated maps in a given cell for each measure, then averaging maps across cells. **(A)** Amplitude of the first IPSC after the light flash, averaged across all cells from WT mice (*N* = 9 cells). **(B)** Amplitude of the first IPSC after the light flash, averaged across all cells from NCAM-null mice (*N* = 12 cells). **(C)** Mean and SEM for first event amplitude as a function of radial distance from the target cell. WT: blue; NCAM-null: red. **(D)** *Z* score for charge following flash averaged across WT cells, from the same cells as in panel (**A**; for calculation, see “Materials and Methods” Section). **(E)** *Z* score for charge following the flash, averaged across cells from NCAM-null mice, from the same cells as in **(B)**. **(F)** Mean and SEM for *Z* score as a function of radial distance from the target cell. *Z* scores were significantly different between genotypes (see text). WT: blue; NCAM-null: red. **(G)** Average probability of input across WT cells as a function of location, based on sites for which the *Z* score was >2.575 (*p* < 0.01). **(H)** Average probability of input across NCAM-null cells, as in panel **(G)**, as a function of location. **(I)** Mean and standard error of the mean for input probability as a function of radial distance. WT: blue; NCAM-null: red. The location of the target cells were aligned at (0,0) in all maps. Asterisk in **(C,F)** indicates *p* < 0.05.

**Figure 8:**
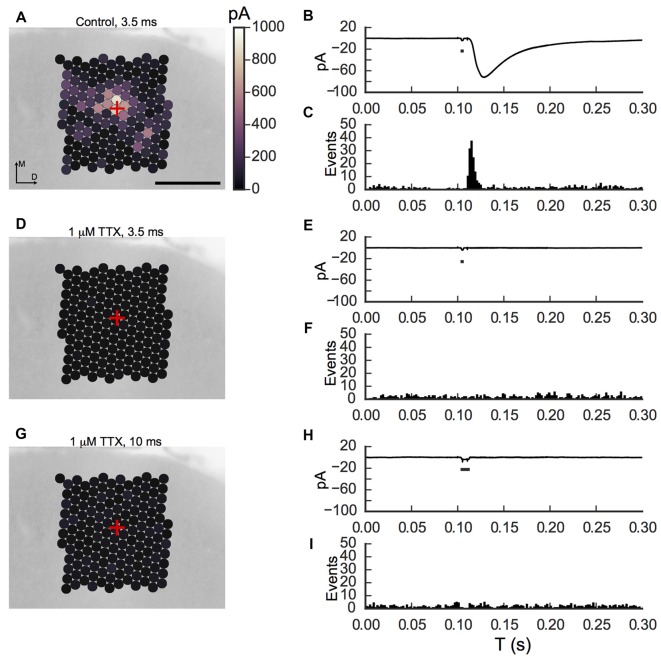
**Tetrodotoxin (TTX) blocks light-evoked responses in pyramidal cells. (A)** Map of average IPSC amplitude onto ACC layer 2/3 pyramidal cell, averaged of three trials for a 3.5 ms light flash. Strong IPSCs were recorded in the vicinity of the cell body (marked by the red cross), and from positions up to 200 μm away. **(B)** Averaged IPSC over all sites in the map, including sites with no evoked responses. The small black bar indicates the timing of the light flash. A stimulus-related artifact is also evident in the trace. **(C)** Peri-stimulus event histogram of all spontaneous and evoked events shows a clear evoked response immediately after the flash. **(D)** Responses were abolished following wash-in of 1 μM TTX (same cell as in **A**). **(E,F)** No current was evident in the average, and there were no events detected in response to the flash in the presence of TTX, although spontaneous miniature IPSCs were still seen. **(G)** A longer flash (10 ms) was tested. **(H,I)** No current was evident in the average, and there were no events detected in response to the flash in the presence of TTX, although spontaneous miniature IPSCs were still seen. Scale bar in **(A)** is 200 μm, and applies to **(D,G)**. M, medial; D, dorsal.

**Figure 9:**
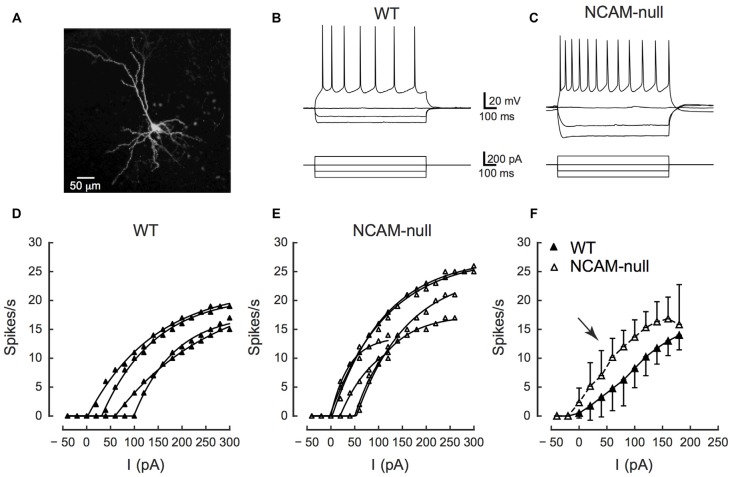
**Comparisons of firing rates of pyramidal cells between WT and NCAM-null mice in ACC in current clamp. (A)** Morphology of a non-EYFP expressing layer 2/3 pyramidal cell. The cell was stained while recording with AlexaFluor 568. **(B)** Pyramidal cell from a WT mouse showing slower firing and an adapting firing (upper traces) in response to intrasomatic current injections (lower traces). **(C)** Pyramidal cell from a NCAM-null mouse showing slow firing and an adapting response (upper traces) to intrasomatic current injections (lower traces). **(D)** Firing frequency as a function of injected current (FI curves) for four pyramidal cells from WT mice. Points are measured firing rates at each current level and the lines are fits for each cell’s FI curve to Equation 1. **(E)** FI curves for six pyramidal cells from NCAM-null mice, plotted in the same manner as panel **(D)**. **(F)** Summary showing mean and SD of FI curves for cells from WT (solid line, filled symbols) and NCAM-null (dashed line, open symbols) mice. The cells from NCAM-null mice showed lower current thresholds, and steeper FI-curves (arrow) than those from WT mice (for details, see Table 2). Calibration bars in **(B)** apply to **(C)**. Recordings were made at 22°C. Error bars in **(F)** are SDs.

**Figure 10:**
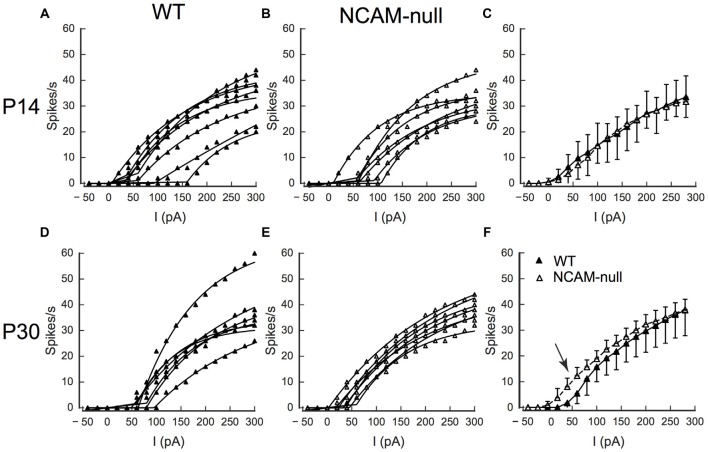
**Intrinsic excitability is increased in NCAM-null pyramidal cells at P30–P34, but not at P14–16. (A)** FI curves for eight WT cells at P14–16. Points show spike rate, lines are fits of Eq. 1 to the FI curves. **(B)** FI curves for seven NCAM-null cells at P14–16. **(C)** Comparison of interpolated FI curves showing no difference in excitability. **(D)** FI curves for seven NCAM-null pyramidal cells at P30–34. **(E)** FI curves for seven NCAM-null pyramidal cells. **(F)** Comparison of interpolated FI curves show lower threshold and increased spike rate for small currents (20–100 pA) in the NCAM-null cells compared to WT cells (arrow). Recordings were made at 34°C, from at least two animals from separate litters of each genotype and age. Error bars in **(C,F)** are SDs.

### Laser Scanning Photostimulation with ChR2

#### Optical Configuration

A 10 mW diode-pumped solid state laser (BWB-10-OEM, B&W Tek) was coupled through a shutter (Uniblitz LS3), to control the duration of excitation from the laser beam for optical stimulation. In most experiments, the laser was then coupled into a 1 m long fiber optic cable (Oz Optics, 50 μm diameter) to additional optics mounted on the back of the microscope. In the experiments examining the effects of TTX, the laser followed a free-space path through turning mirrors and a telescope consisting of a pair of 75 mm lenses. Following the exit from the fiber optic or the focusing telescope, the optical path included (in order) a neutral density filter wheel to control stimulus intensity (NDM4, Thorlabs), a photodiode pickoff to measure beam intensity after the filter wheel, X-Y galvanometer scanning mirrors (Thorlabs), and a second pair of 75 mm scan lenses (Thorlabs). Additionally, a 495 nm dichroic mirror was used to separate the laser illumination from the 530 nm (green) LED light used to image cells filled with dyes. The laser beam underfilled the back focal plane of the objective. The laser light was focused on the slice with a 4× Zeiss objective (0.08NA) so that a small spot, ~50 μm in diameter (measured at the 1/e^2^ intensity positions of the spot), appeared at the surface of the slice. Spot size was computed from measurements of fluorescence from a plastic substrate in the image plane using a CCD camera. Light scattering within the slice will enlarge the area of potential stimulated neurons, although the intensity of the stimulus also decreases with depth. The spatial resolution of the method is best measured empirically by examining the responses of target interneurons (as we present in the results), but is certainly no better than the size of the spot on the surface of the slice. The position of the laser spot was referenced in software against the microscope optical system using the CCD camera, and for registration with images of the cells, the recording site and the slice.

We first measured the effectiveness of the stimulus intensity and duration on individual interneurons to determine the optimal values to use in mapping experiments. To do this, the stimulus intensity was adjusted so that a single action potential was elicited with flashes over the soma in each test interneuron. This occurred when the neutral density filter wheel was rotated to an angle of 110°–120°, corresponding to 0.30–0.44 OD units. The light intensity with these settings was ~40 mW/mm^2^ (averaged over 95% of the area of the spot) at the focal plane, based on the total power measured with a Newport 1971R power meter and an 817 detector, and assuming a beam width of 50 μm. Next, the 473 nm laser was flashed at multiple spots arranged in a hexagonal grid over the interneuron, to map the sensitivity of the cell to stimulation with these parameters. The pickoff photodiode was recorded to monitor and confirm the laser power settings across experiments.

Once the light levels were confirmed to provide focal stimulation of interneurons, we recorded evoked IPSCs from layer 2/3 pyramidal cells in the ACC. To map the spatial organization of presynaptic inputs, individual spots on a hexagonal grid were stimulated at a rate of 1/s. The grid covered the area around the cell, from the top of cortical layer 1 to the bottom of layer 6, and was 0.7–1 mm in width. IPSCs were measured in pyramidal cells in voltage clamp mode with the Cs-based recording solution. Sites were stimulated in a pseudorandom sequence that avoided positions closer than 500 μm in successive trials. Each map was repeated 1–4 times. Multiple trials served to reduce the influence of background activity and allow an assessment of the reliability of the map. The maps indicated the spatial size and magnitude of the local inhibitory circuit that innervates pyramidal cells.

A separate set of experiments examining the effects of TTX was performed using the optical system described above, and the same recording conditions (including the presence of CNQX and D-APV in the recording bath), with the exception that the illumination power was ~60 microwatts, corresponding to ~40–45 mW/mm^2^. Under these conditions, large IPSCs were evoked by stimulating near the postsynaptic pyramidal cell. Recordings were made with CsMeSO_3_-CsCl electrodes under voltage clamp, and small area maps around the target cell soma were made. The maps were repeated three times, and the response at each spot was averaged across the three trials. Laser pulses were 3.5 ms in duration for maps in control solution and in 1 μM TTX, and an additional map was performed in TTX using 10 ms flashes to ensure that responses were blocked with stronger illumination.

### Data Analysis

Data were analyzed with ACQ4 (Campagnola et al., 2014) and custom Python scripts.

For the analysis of firing frequency (*F*) vs. current injection (FI), each cell’s firing rate was the parameterized by fitting to a function of the form:

(1) $$\begin{array}{l} F_{I\leq I_{\text{break}}}=F_{0}+I*F_{1}/I_{\text{break}}\\ F_{I>I_{\text{break}}}=F_{2}*\left(1-\exp\left(-(I-I_{\text{break}})/I_{\text{rate}}\right)\right)+(F_{0}+F_{1}) \end{array}$$

where *F*_0_ is the firing rate with no current (zero for all cells), *F(I)* is the firing rate at current level I (spikes per second), *I*_break_ is the breakpoint between a linear portion of the FI curve and an exponentially rising portion (in units of nA), *F*_1_ is the firing rate at the breakpoint (*I*_break_), *F*_2_ is the dynamic range of the firing rate for currents larger than *I*_break_, and *I*_rate_ is the rate at which the exponentially rising portion of the function changes with current level (units of pA; *I*_rate_ is the current level at which the firing rate rises to 63% (1 − 1/e) of it’s maximal value). Note that *F*_1_/*I*_break_ is the slope of the linear portion of the FI curve below the threshold where firing grows exponentially. Fitting was accomplished using a sequential least squares programming method from the scipy. optimize library (version 0.15.1^2^).

Input resistance was measured as the slope of the IV curve between rest and 20 mV below rest. The membrane time constant was computed as the average time constant of the initial 100 ms of the voltage change in response to hyperpolarizing current pulses that settled to a voltage between 3 and 20 mV negative to rest. The adaptation ratio was measured as the ratio between the mean interspike interval of the last three action potentials and the interspike interval of the first two action potentials during 0.5–1 s long depolarizing current injections that generated at least four action potentials, and which had an average firing rate less than 50 Hz. An adaptation ratio of 1 reflects no adaptation, and an adaptation ratio >1 reflects a slowing of the firing of the cell over time. Discharge patterns were identified and categorized using a scheme adapted for cortical interneurons (Druckmann et al., 2013).

Photostimulation maps were analyzed in a manner similar to previous studies that used laser scanning photostimulation to map responses to glutamate uncaging (Campagnola et al., 2014; Kratz and Manis, 2015). Traces were low-pass filtered, detrended and events identified using an exponential deconvolution method (Richardson and Silberberg, 2008; Campagnola and Manis, 2014). In the experiments testing the effects of TTX, both spontaneous and evoked IPSCs were detected using a template-matching algorithm (Clements and Bekkers, 1997) implemented in Python.

Events in the post-stimulus time window can result from either spontaneous activity or be evoked by presynaptic stimulation. We used a *Z* score calculation based on total charge transfer to determine the likelihood of events being evoked by stimulated presynaptic cells (Barbour and Callaway, 2008). For each map repetition we calculated the total charge transfer from three 30 ms windows: a pre-stimulus baseline region (−40 to −10 ms), a pre-stimulus control region (−70 to −40 ms) and the post-stimulus region (10–40 ms). Laser stimulation took place at 0 ms. We then calculated two *Z* scores: the *Z* score of the post-stimulus region vs. the baseline, and the *Z* score of the pre-stimulus control region vs. the baseline. Scores were calculated by:

(2) $$Z_{\text{spot},\text{post}}=(Qz_{\text{post}}-{\bar{Q}}_{\text{baseline}})/SD(Q_{\text{baseline}})$$

(3) $$Z_{\text{spot},\text{pre}}=(Qz_{\text{pre}}-{\bar{Q}}_{\text{baseline}})/SD(Q_{\text{baseline}})$$

where *Z*_spot,post_ is the *Z* score for the postsynaptic charge for each spot, *QZ*_post_ is the charge measured in the window for each spot, ${\bar{Q}}_{\text{baseline}}$ is the average charge measured across all spots in the map during the baseline, and SD is the standard deviation function. *Z*_spot,pre_ is the *Z* score for the presynaptic window charge for each spot. Sites with an absolute *Z*_spot,post_ >2.575, corresponding to *p* < 0.01, were included as sites with putative presynaptic inputs.

Data shown were derived using three different measures. First, we measured both the peak amplitude, and the charge, of the first event in a 30-ms window following the stimulation, for sites that had a *Z*_spot,post_ >2.575. Second, for these same spots, we used a charge-based analysis (current integrated over time) over a 150 ms post-stimulus window to capture the overall response, which may include both late-firing inputs and the contribution of inputs that fire multiple spikes. Finally, for each cell, we counted the number of times that an input was evoked from each site in the map, to compute the probability of an input arising from each location for the cell. We summarized the data for each cell by averaging measures across repeated maps when available. We then constructed averaged maps for each genotype; these maps included all of the cells mapped in each genotype for which complete maps were available.

### Statistical Analyses

Statistical analyses were performed using Prism (V6.0) and R (V3.1^3^) and the Python package scipy.stats. For comparisons between pairs of independent samples, the Welch’s *t*-test for unequal variances was used (from scipy.stats and Prism, V6.0). Some comparisons were evaluated using 2-sample permutation tests in R with the package “perm” and the two-sample test “permTS”. Other comparisons and the analysis of FI curves used two-way ANOVA as appropriate (Prism V6.0).

## Results

### Characterization of Interneuron Subtypes and NCAM Expression in VGAT-ChR2-EYFP Mouse ACC

To characterize the subtypes of labeled interneurons in the postnatal ACC of VGAT-ChR2-EYFP mice, the expression of GABAergic interneuron markers parvalbumin, calretinin and somatostatin was analyzed by immunofluorescence staining (Figure 1). The vast majority (93%) of EYFP-expressing cells in the cerebral cortex are immunoreactive for GAD67, indicating that this line efficiently labels GABAergic interneurons (Zhao et al., 2011). Of the EYFP positive cells in layer 2/3 of ACC, 48% (SEM 1%) were positive for parvalbumin. Parvalbumin labels basket interneurons, which innervate pyramidal cell soma and proximal dendrites, and chandelier cells, which innervate axon initial segments. Of the EYFP-expressing cells, 15% (SEM 3%) co-labeled with calretinin, a marker of bipolar, bitufted and Cajal-Retzius cells. Somatostatin-positive cells accounted for 10% (SEM 1%) of cells labeled with EYFP. NCAM is produced as a transmembrane glycoprotein that can be modified by polysialylation, or proteolytically cleaved to release the entire extracellular fragment (Maness and Schachner, 2007). To characterize the expression of NCAM and its posttranslational modification by polysialylation in ACC at postnatal (P21) and adult (P40) stages, VGAT-ChR2-EYFP cortical sections were immunostained using antibodies specific for the NCAM-ICD, NCAM-ECD, or polysialylated epitopes of NCAM (PSA-NCAM; Figure 1). Robust expression and colocalization of NCAM ICD and ECD with EYFP were observed at perisomatic puncta (p) and neuropil (n) in the ACC (layer 2/3) at P21, declining to much lower levels in adulthood. These results indicated that NCAM was principally expressed and polysialylated at perisomatic synaptic puncta of EYFP-labeled interneurons, and in the neuropil of the ACC at a postnatal stage (P21) associated with active synaptic remodeling.

### Intrinsic Electrical Excitability of Layer 2/3 Interneurons in ACC

The effectiveness of ChR2 stimulation depends on the expression level and spatial distribution of ChR2 in the membrane, as well as the intrinsic excitability of the ChR2-expressing neurons. ChR2 expression in neuronal membrane is itself not thought to affect the intrinsic excitability of cells in the absence of light (Wang et al., 2007; Zhao et al., 2011). However, deletion of NCAM might result in changes in excitability due to changes in overall circuit activity and subsequent homeostatic adjustments. Therefore, to ensure that there was no change in the excitability of ChR2-expressing neurons due to NCAM deletion, we first compared the intrinsic excitability of ChR2-EYFP-expressing interneurons in current clamp between genotypes. Figure 2A illustrates the typical morphology of fast spiking ChR2-positive interneurons in layer 2/3 of the WT ACC. The responses of interneurons from WT and NCAM-null mice are shown in Figures 2B,C. Layer 2/3 EYFP-expressing cells in WT and NCAM-null mice most frequently showed a fast spiking discharge pattern (11/12 WT cells; one had a slower adapting pattern; 11/13 NCAM cells; one was adapting and one showed a delayed onset firing), and those that were positively identified by filling with a fluorescent dye (5/12 WT, 7/13 NCAM) displayed a non-pyramidal dendritic morphology (Figure 2A). Some cells in each group showed stuttering, delayed, or onset responses for the weakest stimulus levels used. We then used Equation 1 to fit the FI curves for each cell and to obtain estimates of spike threshold, the rate of growth, and maximal firing rate (individual cells in Figures 2D,E). There were no significant differences between the NCAM-null mice and WT mice in any of these parameters (see Table 1 and summary FI curves in Figure 2F). A two way ANOVA of the FI curves demonstrated a significant effect of current (*F*(current)_(17,432)_ = 38.5, *p* < 0.0001), but no effect of genotype (*F*(genotype)_(1,432)_ = 0.43, *P* = 0.51) and no interaction (*F*(interaction)_(17,432)_ = 0.31, *p* = 0.99). No other baseline measures of cell excitability, including resting potential (WT: −58.2 (SD 5.1) mV, *N* = 13; NCAM-null: −58.0 (SD 4.9) mV, *N* = 13; *t*_23.95_ = −0.107, *P* = 0.92), input resistance (WT: 348 (SD 80) MΩ, *N* = 13; NCAM-null: 410 (SD 77) MΩ, *N* = 13; *t*_23.97_ = 1.93, *p* = −0.066), membrane time constant (WT: 11.6 (SD 6.1) ms, *N* = 13; NCAM-null: 18.5 (SD 12.7), *N* = 13; *t*_17.25_ = −1.69, *p* = 0.109), action potential half-width (WT: 0.85 (SD 0.18) ms, *N* = 13; NCAM-null: 1.01 (SD 0.29) ms, *N* = 13; *t*_20.06_ = −1.59, *p* = 0.13), adaptation ratio (WT: 1.47 (SD 0.56), *N* = 13; NCAM-null: 1.42 (SD 0.62), *N* = 13; *t*_23.80_ = 0.207, *p* = 0.84), or maximal firing rate (WT: 73 (SD 21) sp/s, *N* = 13; NCAM-null: 70 (SD 21) sp/s, *N* = 13; *t*_24.0_ = 0.358, *p* = 0.72) were different between the two groups of cells (see Figure 3).

**Table 1:** **Measures of interneuron excitability in wild type (WT) and neural cell adhesion molecule (NCAM)-null mice**.

Parameter	WT (mean, SD) *N* = 10	NCAM-null (mean, SD) *N* = 13	*p*
*F*_0_ (sp/s)	0.00 (SD 0.00)	0.00 (SD 0.00)	1
*I*_break_ (pA)	37.1 (SD 22.6)	21.9 (SD 17.2)	0.097
*F*_1_ (sp/s)	0.002 (SD 0.006)	0.000 (SD 0.000)	0.31
*F*_2_ (sp/s)	64.3 (SD 16.2)	71.3 (SD 19.4)	0.35
*I*_rate_ (pA)	49.7 (SD 18.8)	56.9 (SD 19.3)	0.36

*Cells that exhibited only “*Onset*” patterns were excluded from this analysis. *p* values determined by two-sample permutation test*.

### Responses of Layer 2/3 Interneurons to Photostimulation

We next tested the responses of interneurons to light stimulation, using a 50 μm (1/e^2^ width) spot of 473 nm light. In exploratory experiments, the intensity and duration of the laser flash were varied to find the minimal stimulation that was sufficient to evoke an action potential. Short flashes of 5 ms duration, with the laser light attenuated to ~40 mW/mm^2^ were sufficient to induce a single action potential in most cells. Action potentials were generated either from a single stimulation site, or from a few adjacent sites that were located over and near the cell body. Higher light intensities produced a larger depolarization that could lead to more action potentials, and activate the cell over a larger area, whereas lower intensities were not always sufficient to reach threshold. The spatial organization of light-induced action potentials was evaluated in six WT and eight NCAM-null interneurons from a set of experiments done contemporaneously with the detailed mapping of inputs to pyramidal cells. Example traces and amplitude maps measured under these conditions are shown for two cells from WT mice in Figures 4A1,B1, which illustrates the focal area over which action potentials were elicited; action potentials with latencies from light onset less than 20 ms are colored in blue. Spikes were elicited over one (Figures 4B1,B2) or two (Figures 4A1,A2) spots in most cells. The area surrounding the spike eliciting sites evoked subthreshold depolarization (visible as blue depolarizations, and colored spots in the amplitude maps) or no evoked response. Spontaneous synaptic events gave rise to occasional small depolarizations as indicated by the scattered depolarizations and the brown spots far from the recorded interneuron in all maps. Interneurons in NCAM-null mice showed a similar pattern of activation in response to the same light stimulation, as illustrated for two cells in Figures 4C1,C2,D1,D2. Occasionally, spontaneous spikes also occurred outside the response measurement window (gray spikes in Figures 4A1,D1). To summarize, all (6/6) WT cells tested responded to laser stimulation at this level with at least one spot producing a single light-evoked spike, whereas 8/10 interneurons from NCAM-null mice tested responded with at least one spot with a single spike. The other two cells from NCAM-null mice showed light-evoked depolarization that did not reach spike threshold. The latencies of evoked spikes were not different between the two genotypes, for either the shortest latency sites in each tested cell (WT: 6.9 ms (SD 3.1, range 3.5–11.1, *N* = 6 cells); NCAM-null 9.1 ms (SD 2.7, range 5.0–13.3, *N* = 8 cells); *p* = 0.183, two-sample permutation test), nor for all evoked spikes (WT: 9.7 ms (SD 2.1 ms, range 6.1–11.9 ms, *N* = 6 cells); NCAM-null 12.7 ms (SD 4.1 ms, range 8.2–20.3 ms, *N* = 8 cells), *p* = 0.127, two-sample permutation test). The optimal stimulus site(s) were at or close to the interneuron cell body, suggesting that action potentials were generated by stimulation over the cell bodies and proximal axons and dendrites of the interneurons, rather than from stimulation of their distal dendritic processes or distal axons (also see Kätzel et al., 2011). We conclude that focused laser stimulation for 5 ms at 40 mW/mm^2^ with a ~50 μm spot in the WT and NCAM-null mice crossed with the VGAT-ChR2-EYFP mice is sufficient to evoke single action potentials in most superficial interneurons, and that action potentials are evoked for light delivered over a limited area of approximately the size of the illuminating spot, near or at the soma, and with a short (7–13 ms) latency.

### Mapping Inhibitory Inputs to Layer 2/3 Pyramidal Cells in ACC

Once we determined the necessary stimulation conditions for the interneurons, we proceeded to characterize the functional connections from interneurons to pyramidal cells. To do this, we mapped the spatial distribution of stimulation sites that produced IPSCs (in voltage clamp) in layer 2/3 pyramidal cells in ACC. The WT group (*N* = 9 cells) was stimulated with an average power of 42.1 (SD = 1.7) mW/mm^2^ (*N* = 9), with a mean spot size of 49 μm (SD = 1.6), whereas the NCAM-null group (*N* = 12 cells) was stimulated with an average of 42.7 mW/mm^2^ (SD = 5.2) and a mean spot size of 52 μm (SD = 3.4).

Example IPSCs recorded in response to photostimulation are shown in Figures 5A–E. Light-evoked postsynaptic responses were measured in voltage clamp at −70 mV in the presence of antagonists of excitatory receptors, and using an intracellular solution with a high chloride concentration (see “Materials and Methods” Section), which resulted in easily-detected, inward IPSCs that were uncontaminated by excitatory postsynaptic currents. Responses occurred within ~20 ms of the laser flash (see also Figure 8) and individual evoked events had a stereotypical shape with a fast rise and slower falling phase. In some trials, multiple events were evident (Figures 5B,D), suggesting either that the light stimulation produced short bursts of action potentials in the interneurons or activated multiple cells.

Maps of inhibitory inputs to pyramidal cells were constructed from responses to light stimulation over an area 0.7–1.0 mm wide (centered on the recording site) by ~1.2 mm deep, covering all layers in the ACC, interrogating approximately 500 sites. Data were analyzed in nine cells from four WT mice (two cells each from three mice, and three cells from one mouse), and in 12 cells from 7 NCAM-null mice (two cells each from five mice, and one cell each from the remaining two mice). Figure 6 shows example maps with traces (panels **A1,B1,C1,D1**) and of charge transferred during the first evoked event following the light flash (panels **A2,B2,C2,D2**) for both genotypes, superimposed on the slice. These panels show single maps from individual cells. Most of the inhibitory input arose from regions close to the recorded cell body, although scattered events were seen from sites up to 700 μm away from the target cell. Some of these distant IPSCs may have arisen from spontaneous spiking or spontaneous quantal release in interneurons in the slice, as they were largely observed only in single trials when maps were repeated three times (six WT and five NCAM-null cells) or four times (one NCAM-null cell; the remaining cells had only one or two maps so that the difference between spontaneous and evoked events could not be as clearly delineated). Such spontaneous events cannot be assigned as arising from a specific position in the map. Individual evoked events had a stereotypical shape with a fast rise and slower falling phase, and in some cases the presence of multiple evoked responses in succession suggests that the stimulation had resulted in multiple action potentials or in activation of multiple cells. Similar input patterns were seen in cells from the NCAM-null mice (Figures 6C1,C2,D1,D2).

To evaluate the effects of the NCAM deletion, we then averaged maps across cells using a cell-centered coordinate system for each genotype, based on first event amplitude, the *Z* score (see “Materials and Methods” Section), and the probability of observing evoked IPSCs at each site. The summary maps are presented in Figure 7. Figures 7A,B show the amplitude of the first post-flash IPSC for WT and NCAM-null mice, respectively. The response in the NCAM-null mice appears less scattered and is more focused within about 200 μm of the soma. To be sure that different laminar sampling between the WT and NCAM-null mice does not influence the spatial differences, we measured the distance from the each cell to the pial surface and the L1 to L2/3 boundary. The mean cell depth from the pial surface was not different for the WT and NCAM-null cells (WT: 278 (SD = 48) μm, *N* = 9, NCAM-null: 257 (SD = 64) μm, *N* = 12; *t*_19.0_ = 0.891, *p* = 0.38). Likewise, the distance from the L1 to L2 boundary was not different (WT: 128 μm (SD = 47), NCAM-null: 129 μm (SD 64), *t*_19.0_ = 0.0012, *p* = 0.999).

Figure 7C compares the amplitudes of the inputs within a 200 μm radius of the cell body, in 50 μm bins. The average first-event amplitude than from the NCAM-null cells appears to be consistently larger than that of the WT cells for the closest site. A two-way ANOVA over all distances, comparing genotype × distance yielded (*F*(genotype)_(1,361)_ = 0.39, *P* = 0.53; *F*(distance)_(18,361)_ = 8.14 *P* < 0.0001; *F*(interaction)_(18,361)_ = 0.58, *P* = 0.53). A post-test comparing genotypes across distances shows that for sites within a 50 μm radius of the recorded cell, the first event amplitude was larger in NCAM-null cells than in WT cells (*P* < 0.05; Sidak multiple comparison test).

The second row (Figures 7D,E) shows the *Z* scores, based on the charge measured over 30 ms windows during the baseline and 30 ms immediately after the stimulus; these scores normalize the responses against the spontaneous activity in individual cells. The spatial patterns are similar to those in the top row (compare Figures 7A,B,D,E). However, here the distinction between responses from the cells from the WT and NCAM-null mice was clearer (Figure 7F). A two-way ANOVA revealed a significant effect of both distance and genotype (*F*(genotype)_(1,355)_ = 4.60, *P* = 0.033; *F*(distance)_(18,355)_ = 8.18, *P* < 0.0001; *F*(interaction)_(18,355)_ = 1.32, *P* = 0.17). A post-test comparing genotypes across distances shows that for the closest bin, the *Z* score was larger in NCAM-null cells than in WT cells (*P* < 0.05; Sidak multiple comparison test). Although the mean values are also larger at other distances, the differences are not significant. The average charge during the spontaneous window (pre-stimulus) was not different between the WT and NCAM-null cells (WT: 0.063, SD = 0.33 pC; NCAM-null: 0.058, SD = 0.051 pC, *p* = 0.82, 2-sample permutation test), which excludes the possibility that the *Z* score is biased by the presence of spontaneous events occurring at different rates in the two genotypes, rather than by the light evoked responses.

Finally, we computed the probability of responses at each site across cells. The probability that a response at a given site for a given cell was computed using the *Z* score and a cutoff value of 2.575 (*p* < 0.01), so that each cell had a probability map consisting of 0’s and 1’s. These maps were then averaged across cells, and are summarized in the third row (Figure 7G: WT, Figure 7H: NCAM-null). Again, the maps show a qualitative difference, and the mean values for the cells from the NCAM-null mice are consistently larger than those of the WT mice (Figure 7I). A two-way ANOVA reveals only an effect of distance, but not of genotype (*F*(genotype)_(1,355)_ = 2.36, *P* = 0.13; *F*(distance)_(18,355)_ = 11.2, *P* < 0.0001; *F*(interaction)_(18,355)_ = 0.71, *P* = 0.80). Post-tests revealed no difference between genotypes.

One concern about the mapping experiments is that the axons of cells expressing ChR2 could be directly excited by optical stimulation. Although our experiments were performed under conditions where a weak photic stimulus was sufficient to drive action potentials when delivered over the soma (Figure 4), but not elsewhere on the cell, it remains possible that depolarization elicited elsewhere, but not observable at the cell body, could still lead to transmitter release. To partially address this issue, we performed experiments where we blocked action potential initiation with 1 μM TTX. Maps and traces from an example cell are summarized in Figure 8. Figure 8A shows a response map recorded in a pyramidal cell from a WT VGAT-ChR2-EYFP mouse. Optical stimulation from the area surrounding the target cell with a 3.5 ms duration laser pulse (60 μW, 40–45 mW/mm^2^) produces large IPSCs. Figure 8B shows the averaged IPSC from all sites in the map. The peri-stimulus time histogram of the onset times for both spontaneous and photically-elicited events is shown in Figure 8C. Although a background of spontaneous events was evident, there was a clear clustering of responses between 10 ms and 25 ms after the flash onset. After the slice was incubated with 1 μM TTX, no responses were observed for either a 3.5 ms (Figures 8D–F) or 10 ms duration light flash (Figures 8G–I) from the same cell. Similar results were observed for two other cells before and after TTX, and in one additional cell tested only in TTX (where we previously obtained light-evoked responses in another cell in the same slice); thus in four cells we confirmed that under our experimental conditions the laser flashes did not evoke detectable release from the interneurons. From these experiments, we conclude that depolarization-evoked action potentials are required to produce the IPSCs, and that depolarization of terminals by ChR2 alone is not sufficient. Additional caveats regarding this conclusion are explored in the discussion.

We conclude that stronger inhibition (as measured by the first event amplitude and the total charge of evoked events) is evoked for sites near the pyramidal cell body in the cells from the NCAM-null mice, and that the inhibition is evoked by stimulating in a region located slightly below the cell.

### Excitability of Pyramidal Cells in NCAM-null ACC

We compared the intrinsic excitability of layer 2/3 pyramidal cells in the ACC between the WT and NCAM-null mice in two sets of experiments. The first set of experiments were performed under the same conditions used in the mapping studies, at 22°C, in mice aged P30–P40. Figure 9A shows an example of an AlexaFluor 568-stained pyramidal cell, along with responses to current steps from WT and NCAM-null mice (Figures 9B,C). The increase of firing rate with current was steeper in NCAM-null than in WT mice (individual cells shown in Figure 9D,E; summarized in Figure 9F). A two way ANOVA of the FI curves demonstrated a significant effect of current (*F*(current)_(12,117)_ = 41.9, *p* < 0.0001), and of genotype (*F*(genotype)_(1,117)_ = 35.1, *p* < 0.0001), but no interaction (*F*(interaction)_(17,117)_ = 1.25, *p* = 0.26). Consistent with this, the value of *I*_rate_ was smaller in the NCAM-null mice (see Table 2) indicating that for a given value of injected current, the cells from NCAM-null mice fire at a higher rate than in WT mice. Except for input resistance (WT: 289 (SD = 41) MΩ, *N* = 4; NCAM-null: 410 (SD = 68) MΩ, *N* = 7; *t*_5.72_ = −3.305, *p* = 0.0096), none of the other measures of cell excitability were significantly different between the two groups of cells, including resting potential (WT: −66.9 (SD = 3.0) mV, *N* = 4; NCAM-null: −63.7 (SD = 2.9) mV, *N* = 7; *t*_5.48_ = −1.530, *p* = 0.18), maximal firing rate (WT: 42 (SD = 3) Hz, *N* = 4; NCAM-null: 40 (SD = 10) Hz, *N* = 7; *t*_4.08_ = 0.5543, *p* = 0.59), action potential half-width (WT: 2.83 (SD = 0.60) ms, *N* = 4; NCAM-null: 2.97 (SD = 0.62) ms, *N* = 7; *t*_5.64_ = −0.3415, *p* = 0.74) or adaptation ratio (WT: 3.18 (SD = 0.51), *N* = 4; NCAM-null: 2.26 (SD = 0.71), *N* = 7; *t*_5.98_ = 2.248, *p* = 0.056).

**Table 2:** **Measures of pyramidal cell excitability in WT and NCAM-null mice at 22–25°C**.

Parameter	WT (mean, SD) *N* = 4	NCAM-null (mean, SD) *N* = 7	*p*
*F*_0_ (sp/s)	0.00 (SD 0.00)	0.00 (SD 0.00)	1
*I*_break_ (pA)	43.9 (SD 29.3)	21.6 (SD 25.1)	0.31
*F*_1_ (sp/s)	0.001 (SD 0.003)	0.000 (SD 0.000)	0.36
*F*_2_ (sp/s)	18.6 (SD 5.1)	24.0 (SD 2.0)	0.54
*I*_rate_ (pA)	169.3 (SD 52.1)	68.5 (SD 28.8)	0.036

*p values determined by two-sample independent t-test*.

These results suggest that the pyramidal cells in the NCAM-null mice may have increased intrinsic excitability, in that they will fire at a higher rate in response to a current injection than cells from their WT counterparts. To explore this further, we compared the excitability of the ACC pyramidal cells in between mice at P14–16 (prior to the primary period of inhibitory synapse pruning) and at P30–34 when the NCAM-null mice exhibit an elevated number of perisomatic inhibitory puncta (Brennaman et al., 2013). In these experiments, recordings were made at 34°C to better reflect excitability at closer to physiological temperature. At P14–16, there was no difference in the excitability of L2/3 pyramidal cells (Figures 10A–C) as assessed by analysis of the individual FI curves. A two way ANOVA of the FI curves (Figure 10C) demonstrated a significant effect of current (*F*(current)_(17,234)_ = 51.3, *p* < 0.0001), but no effect of genotype (*F*(genotype)_(1,234)_ = 0.38, *p* = 0.54), but no interaction (*F*(interaction)_(17,234)_ = 0.11, *p* = 1.0). Neither were any differences detected in resting potential (WT: −61.9 (SD = 6.7) mV, *N* = 8; NCAM-null: −65.0 (SD = 5.7) mV, *N* = 7; *t*_13.88_ = 0.8838, *p* = 0.39), input resistance (WT: 289 (SD = 102) MΩ, *N* = 8; NCAM-null: 306 (SD = 87) MΩ, *N* = 7; *t*_13.90_ = −0.3330, *p* = 0.74), membrane time constant (WT: 19.7 (SD = 9.4) ms, *N* = 8; NCAM-null: 20.2 (SD = 8.4) ms, *N* = 7; *t*_13.97_ = −0.102, *p* = 0.92), maximal firing rate (WT: 70 (SD = 14) Hz, *N* = 8; NCAM-null: 66 (SD = 10) Hz, *N* = 7; *t*_13.34_ = 0.631, *p* = 0.54), or adaptation ratio (WT: 2.49 (SD = 0.70), *N* = 8; NCAM-null: 3.97 (SD = 1.65), *N* = 7; *t*_9.15_ = −2.05, *p* = 0.075). The action potential half-width was significantly wider in the NCAM-null mice at this age (WT: 0.95 ms, SD = 0.17, *N* = 8; NCAM-null: 1.42 ms, SD = 0.23, *N* = 7; *t*_12.42_ = −4.046, *p* = 0.002).

However, at P30–34, cells in the NCAM-null mice began firing at lower current levels than cells from WT mice (Figures 10D–F). A two way ANOVA of the FI curves (Figure 10F) demonstrated a significant effect of current (*F*(current)_(17,216)_ = 98.3, *p* < 0.0001), and of genotype (*F*(genotype)_(1,216)_ = 12.7, *p* = 0.0005), but no interaction (*F*(interaction)_(17,216)_ = 0.53, *p* = 0.94). Again, there were no differences in resting membrane potential (WT: −64.7 (SD = 3.6) mV, *N* = 7; NCAM-null: −63.0 (SD = 4.2) mV, *N* = 7; *t*_11.76_ = −0.741, *p* = 0.47), input resistance (WT: 270 (SD = 50) MΩ, *N* = 7; NCAM-null: 291 (SD = 34) MΩ, *N* = 7; *t*_10.59_ = −0.869, *p* = 0.40), membrane time constant (WT: 14.0 (SD = 4.9) ms, *N* = 7; NCAM-null: 20.5 (SD = 9.8) ms, *N* = 7; *t*_8.79_ = −1.454, *p* = 0.18), maximal firing rate (WT: 75 (SD = 20) Hz, *N* = 7; NCAM-null: 76 (SD = 8) Hz, *N* = 7; *t*_7.92_ = −0.066, *p* = 0.95), action potential half-width (WT: 1.06 (SD = 0.21) ms, *N* = 7; NCAM-null: 0.879 (SD = 0.124) ms, *N* = 7; *t*_9.84_ = 1.851, *p* = 0.09), or adaptation ratio (WT: 3.66 (SD = 1.84), *N* = 7; NCAM-null: 3.65 (SD = 1.57), *N* = 7; *t*_11.71_ = 0.015, *p* = 0.99). To summarize, pyramidal cells in P30–34 NCAM-null mice at 34°C showed a higher firing rate for currents just above threshold than seen in WT cells, similar to the effect seen at 22°C.

## Discussion

We found that NCAM deletion increased the strength of close-in inhibition from interneurons to layer 2/3 pyramidal cells in the ACC. This is consistent with the increase of GABAergic perisomatic synaptic puncta previously observed following deletion of NCAM (Pillai-Nair et al., 2005; Brennaman et al., 2013). There was no evidence for an increase in the strength of quantal events, as evaluated by the average spontaneous charge in the absence of stimulation. We observed no difference in the excitability of the interneurons, suggesting that circuit effects did not produce homeostatic changes in intrinsic excitability. However, we did observe an increase in excitability of the postsynaptic pyramidal cells from NCAM-null mice when compared to WT mice, possibly indicating a compensatory response to an increase in inhibitory tone.

We used laser-scanning photostimulation of ChR2-expressing GABAergic interneurons to evaluate the organization and strength of inhibitory inputs to pyramidal cells. There are several considerations that must be recognized when interpreting results obtained with this method. First, optical stimulation offers only limited control over the spiking patterns of the ChR2-expressing cells. The intensity of the stimulating light and its flux across channels in the membrane is a complex, but largely decreasing, function of depth within the slice. Thus, individual cells can be stimulated in different ways: either by intense illumination of a small population of ChR2 molecules in a limited part of the cell, or by more diffuse illumination over a larger area of the membrane. As a result, the time course of depolarization, and the efficacy of the depolarization to elicit spikes will vary. For this reason, our analysis of the optically elicited response included measures of the synaptic charge (current over time) over a longer time period. However, this also means that, in any given experiment, maps based on near-threshold optical stimulation will represent only a subset of the inputs to a target cell (e.g., not the full connectivity). Second, the sensitivity of cells to light stimulation must be evaluated for each genotype and expression method. Our measurements suggest that there is no difference in average sensitivity to light stimulation between the two genotypes used in this study, which allows the responses to optical stimulation to be directly compared when matched stimulus energies are used. However it is also clear that the sensitivity of individual cells varies, and with a fixed illumination level not all recorded interneurons could be driven to spike threshold. Third, the VGAT promoter can result in expression in a variety of cell types. In layer 2/3 of the ACC of the mice used here, the expression of ChR2 and EYFP appears in the parvalbumin-positive cells, somatostatin-positive cells, and calretinin-positive cells (Figure 1). More precisely targeted expression of ChR2 would be needed to restrict stimulation to particular subclasses of cortical interneurons. Given that the largest fraction of VGAT-positive cells were also labeled for parvalbumin (48%), and that an increase in perisomatic innervation was seen previously in pyramidal cells from NCAM-null mice, a somatically-targeted inhibition from basket cells is likely to contribute to the increased functional inhibition seen in the present study. However, our experiments do not exclude that inhibitory synapses from other classes of interneurons also contribute to the increased functional inhibition onto pyramidal cells. A final consideration is that the lateral spatial resolution of our stimulus is no smaller than 50 μm, which limits the localization of the interneurons to that scale. This spot size, and the use of single-photon stimulation, does not yield single-cell resolution. More precise localization of stimulated cells is not possible to achieve experimentally with our stimulation because of light scattering, the approximately Gaussian shape of the illuminated spot in the plane of the slice, and the unknown effective depth of penetration of light into the tissue. Two-photon optical stimulation (with either glutamate uncaging or ChR2 stimulation), potentially coupled with paired recording, could provide more precise localization (Fino and Yuste, 2011; Packer and Yuste, 2011). However, single photon laser scanning photostimulation on the scale that we have used appears to be an efficient way to obtain an overview of local connectivity.

One concern is that it has been demonstrated that optical stimulation can cause direct release from axon terminals (Petreanu et al., 2007, 2009). These prior observations are largely made in cells expressing high levels of ChR2 using viral promoters, in the presence of potassium channel blockers, and where information is available, using significantly higher illumination power densities than utilized for perisomatic stimulation in interneurons. We show that under our stimulus conditions no evoked responses can be seen when sodium channels are blocked with TTX. This suggests that action potential initiation is necessary for transmitter release, and that the depolarization of terminals by light activation of ChR2 is not sufficient for release. Furthermore, with the low light power used here, we found that we could elicit action potentials from single sites located over the soma, without seeing evidence for action potentials from other sites in the tissue. Taken together these observations suggest that using LSPS can be used to selectively stimulate neurons at their cell bodies without stimulating dendrites or proximal axons under appropriate conditions, which include using a small illumination spot and minimal illumination power (Kätzel et al., 2011; Kim et al., 2014; Brill et al., 2016). A caveat to this interpretation is that we cannot definitely rule out the possibility that optical stimulation produces action potentials in axon terminals that cause release (and can be blocked by TTX), but which fails to antidromically propagate into the cell body. Therefore, although our maps certainly reflect the locations of presynaptic cells, they may also be contaminated in part by direct responses of axons or terminals in the vicinity of the target cell.

Our results are in general agreement with direct measurements of interneuron to pyramidal cell connectivity made using 2-photon stimulation and paired recording methods (Fino and Yuste, 2011; Packer and Yuste, 2011; Jiang et al., 2015). The spatial domain over which we observe the main input sources falls off with a space constant of 100–120 μm, consistent with the dense connectivity within 200 μm reported by Packer and Yuste (2011) for parvalbumin-positive interneurons (basket and chandelier cells), and Fino and Yuste (2011) for somatostatin-expressing interneurons. We observed a tendency for inputs to the layer 2/3 cells to arise from sites below the target cell, whereas Packer and Yuste (2011) observed a tendency for inputs to arise from above the target cell in frontal cortex and layer 2/3 of somatosensory cortex. These differences may result from different sampling biases of the location of the recorded cells in the two studies. Inspection of our recordings shows that they tended to be from cells in the upper part of layer 2/3 (mean distance from the pia of 260–280 μm), and because there are few cells in layer 1, the primary sources of inhibitory input will arise from cells located deep to the recorded cell.

There are two main conclusions that we can draw from the present measurements. First, the optically-evoked synaptic responses were on average larger in the NCAM-null mice for sites that were close to the cell body. The larger responses could result from increased quantal size, increased quantal content, higher release probability, more exuberant connections from individual presynaptic cells, or a higher density of presynaptic cells that synapse onto the target cells. It is difficult to disentangle these possibilities without direct measures of release probability from individual cells. The eIPSC amplitude, on average, is *P*_r_*N_t_*N_pre_*I_r_, where *P*_r_ is the release probability [0,1], *N*_t_ is the average number of terminals from a single cell, *N*_pre_ is the number of presynaptic cells, and *I*_r_ is the current produced by a release from a single terminal under quiescent conditions. Our previous measurements indicate that quantal size of GABAergic events in anterior cingulate layer 2/3 cells was larger by about 16% in the NCAM-null mice (Brennaman et al., 2013). However, here we observed that the total charge during the baseline window, which depends on IPSP amplitude and time course, as well as the spontaneous event rate, was not different between genotypes. The larger number of synaptic terminals observed in slice cultures (Brennaman et al., 2013) could represent an increase in the convergence of presynaptic cells with the same number of terminals per cell, or an increase in the number of terminals from individual presynaptic cells. Regardless, our results indicate that this anatomically observed increase in terminals is accompanied by an increase in functional inhibition onto the pyramidal cells.

The interneurons in this study showed lower firing rates and slightly higher input resistances than reported in studies performed at elevated temperatures (Halabisky et al., 2006; Helmstaedter et al., 2009), but are consistent with published measurements made at room temperature (Fino and Yuste, 2011). Although the interneurons did not show any difference in excitability between the two genotypes, pyramidal cells from the NCAM-null mice showed an increased rate of firing at low current levels, without any other changes in input resistance, time constant, resting potential, or action potential shape. The increased excitability was observed in two independent sets of experiments performed at both 22°C and 34°C. The increase in firing rate in pyramidal cells is consistent with the existence of a homeostatic excitability set point that attempts to maintain firing rates given long-term changes in the average input. In the presence of an increased inhibitory tone resulting from the exuberant inhibitory synapses in the NCAM-null mice, the cells could seek to maintain an appropriate balance of excitation and inhibition in the circuit by increasing their sensitivity to depolarization. Similar examples of increased intrinsic excitability have been seen in the visual and auditory cortical areas following sensory deprivation (Rao et al., 2010; Lambo and Turrigiano, 2013) Although it has not been directly evaluated, the strength of excitatory synapses might also be predicted to be increased in the layer 2/3 pyramidal cells of NCAM-null mice.

Inhibitory interneurons, and particularly basket cells and other parvalbumin-expressing neurons in the cortex, play a crucial role in the generation of cortical gamma oscillations in the 40–80 Hz range (Gonzalez-Burgos et al., 2015), and gamma oscillations are thought be critical in cognitive function and feature binding in sensory systems (Fries, 2009). Modeling studies indicate that the generation of gamma oscillations depends on the strength and timing of inhibition in the network (Economo and White, 2012; Kuki et al., 2015; Tikidji-Hamburyan et al., 2015), the presence of asynchronous release from inhibitory neurons (Volman et al., 2011), as well as the excitability of both excitatory and inhibitory cells (Baroni et al., 2014). Changes in any of these factors can affect the period of oscillations, as well as the duration of oscillatory episodes. Our observations show that inhibition is stronger in NCAM-null mice, and would be predicted to slow and possibly desynchronize gamma oscillations. Such effects of the dysregulation of NCAM on gamma oscillations could contribute to the cognitive and sensory deficits associated with schizophrenia, autism and bipolar disorder.

## Author Contributions

PFM and PBM designed research. XZ, CSS, MBK, PBM and MRK performed electrophysiological experiments. MBK and PBM wrote analysis scripts. MBK, XZ and PBM analyzed electrophysiological data. CSS performed and analyzed immunolabeling experiments. XZ, MBK, CSS, PFM and PBM wrote the manuscript.

## Conflict of Interest Statement

The authors declare that the research was conducted in the absence of any commercial or financial relationships that could be construed as a potential conflict of interest.

## Footnotes

^1^http://antibodyregistry.org/

^2^www.scipy.org

^3^www.r-project.org

## Abbreviations

ACC: anterior cingulate cortex
ACSF: artificial cerebrospinal fluid
ChR2: channelrhodopsin-2
CNQX: 6-cyano-7-nitroquinoxaline-2,3-dione
D-APV: D-2-amino-5-phosphonovalerate
EYFP: enhanced yellow fluorescent protein
E/I: excitatory/inhibitory
FI: frequency-current
GABA: gamma-amino butyric acid
IPSC: inhibitory postsynaptic current
LED: light emitting diode
NCAM: neural cell adhesion molecule
SD: standard deviation
TTX: Tetrodotoxin
VGAT: vesicular GABA transporter
WT: wild type.

## References

[B1] Anney R. Klei L. Pinto D. Regan R. Conroy J. Magalhaes T. R.. (2010). A genome-wide scan for common alleles affecting risk for autism. Hum. Mol. Genet. 19, 4072–4082. 10.1093/hmg/ddq307 20663923 PMC2947401

[B2] Arai M. Itokawa M. Yamada K. Toyota T. Arai M. Haga S.. (2004). Association of neural cell adhesion molecule 1 gene polymorphisms with bipolar affective disorder in Japanese individuals. Biol. Psychiatry 55, 804–810. 10.1016/j.biopsych.2004.01.009 15050861

[B3] Atz M. E. Rollins B. Vawter M. P. (2007). NCAM1 association study of bipolar disorder and schizophrenia: polymorphisms and alternatively spliced isoforms lead to similarities and differences. Psychiatr. Genet. 17, 55–67. 10.1097/ypg.0b013e328012d850 17413444 PMC2077086

[B4] Ayalew M. Le-Niculescu H. Levey D. F. Jain N. Changala B. Patel S. D.. (2012). Convergent functional genomics of schizophrenia: from comprehensive understanding to genetic risk prediction. Mol. Psychiatry 17, 887–905. 10.1038/mp.2012.37 22584867 PMC3427857

[B5] Barbour D. L. Callaway E. M. (2008). Excitatory local connections of superficial neurons in rat auditory cortex. J. Neurosci. 28, 11174–11185. 10.1523/JNEUROSCI.2093-08.2008 18971460 PMC2610470

[B6] Baroni F. Burkitt A. N. Grayden D. B. (2014). Interplay of intrinsic and synaptic conductances in the generation of high-frequency oscillations in interneuronal networks with irregular spiking. PLoS Comput. Biol. 10:e1003574. 10.1371/journal.pcbi.1003574 24784237 PMC4006709

[B7] Bartley A. F. Huang Z. J. Huber K. M. Gibson J. R. (2008). Differential activity-dependent, homeostatic plasticity of two neocortical inhibitory circuits. J. Neurophysiol. 100, 1983–1994. 10.1152/jn.90635.2008 18701752 PMC2576194

[B8] Bartos M. Vida I. Jonas P. (2007). Synaptic mechanisms of synchronized gamma oscillations in inhibitory interneuron networks. Nat. Rev. Neurosci. 8, 45–56. 10.1038/nrn2044 17180162

[B9] Brennaman L. H. Zhang X. Guan H. Triplett J. W. Brown A. Demyanenko G. P.. (2013). Polysialylated NCAM and ephrinA/EphA regulate synaptic development of GABAergic interneurons in prefrontal cortex. Cereb. Cortex 23, 162–177. 10.1093/cercor/bhr392 22275477 PMC3513957

[B10] Brill J. Mattis J. Deisseroth K. Huguenard J. R. (2016). LSPS/optogenetics to improve synaptic connectivity mapping: unmasking the role of basket cell-mediated feedforward inhibition. eNeuro 3:e0142-15.2016. 10.1523/ENEURO.0142-15.2016 27517089 PMC4976301

[B11] Calandreau L. Márquez C. Bisaz R. Fantin M. Sandi C. (2010). Differential impact of polysialyltransferase ST8SiaII and ST8SiaIV knockout on social interaction and aggression. Genes Brain Behav. 9, 958–967. 10.1111/j.1601-183X.2010.00635.X 20659171

[B13] Campagnola L. Kratz M. B. Manis P. B. (2014). ACQ4: an open-source software platform for data acquisition and analysis in neurophysiology research. Front. Neuroinform. 8:3. 10.3389/fninf.2014.00003 24523692 PMC3906568

[B12] Campagnola L. Manis P. B. (2014). A map of functional synaptic connectivity in the mouse anteroventral cochlear nucleus. J. Neurosci. 34, 2214–2230. 10.1523/JNEUROSCI.4669-13.2014 24501361 PMC3913869

[B14] Caroni P. Chowdhury A. Lahr M. (2014). Synapse rearrangements upon learning: from divergent-sparse connectivity to dedicated sub-circuits. Trends Neurosci. 37, 604–614. 10.1016/j.tins.2014.08.011 25257207

[B15] Casey J. P. Magalhaes T. Conroy J. M. Regan R. Shah N. Anney R.. (2012). A novel approach of homozygous haplotype sharing identifies candidate genes in autism spectrum disorder. Hum. Genet. 131, 565–579. 10.1007/s00439-011-1094-6 21996756 PMC3303079

[B16] Cerkevich C. M. Qi H.-X. Kaas J. H. (2013). Thalamic input to representations of the teeth, tongue, and face in somatosensory area 3b of macaque monkeys. J. Comp. Neurol. 521, 3954–3971. 10.1002/cne.23386 23873330 PMC3893768

[B17] Chen N. Sugihara H. Sur M. (2015). An acetylcholine-activated microcircuit drives temporal dynamics of cortical activity. Nat. Neurosci. 18, 892–902. 10.1038/nn.4002 25915477 PMC4446146

[B18] Chu J. Anderson S. A. (2015). Development of cortical interneurons. Neuropsychopharmacology 40, 16–23. 10.1038/npp.2014.171 25103177 PMC4262895

[B19] Clements J. D. Bekkers J. M. (1997). Detection of spontaneous synaptic events with an optimally scaled template. Biophys. J. 73, 220–229. 10.1016/s0006-3495(97)78062-7 9199786 PMC1180923

[B20] Cremer H. Lange R. Christoph A. Plomann M. Vopper G. Roes J.. (1994). Inactivation of the N-CAM gene in mice results in size reduction of the olfactory bulb and deficits in spatial learning. Nature 367, 455–459. 10.1038/367455a0 8107803

[B21] Demyanenko G. P. Tsai A. Y. Maness P. F. (1999). Abnormalities in neuronal process extension, hippocampal development, and the ventricular system of L1 knockout mice. J. Neurosci. 19, 4907–4920. 10366625 10.1523/JNEUROSCI.19-12-04907.1999 PMC6782672

[B22] Druckmann S. Hill S. Schürmann F. Markram H. Segev I. (2013). A hierarchical structure of cortical interneuron electrical diversity revealed by automated statistical analysis. Cereb. Cortex 23, 2994–3006. 10.1093/cercor/bhs290 22989582

[B23] Economo M. N. White J. A. (2012). Membrane properties and the balance between excitation and inhibition control gamma-frequency oscillations arising from feedback inhibition. PLoS Comput. Biol. 8:e1002354. 10.1371/journal.pcbi.1002354 22275859 PMC3261914

[B24] Fino E. Yuste R. (2011). Dense inhibitory connectivity in neocortex. Neuron 69, 1188–1203. 10.1016/j.neuron.2011.02.025 21435562 PMC3086675

[B25] Freund T. F. Katona I. (2007). Perisomatic inhibition. Neuron 56, 33–42. 10.1016/j.neuron.2007.09.012 17920013

[B26] Fries P. (2009). Neuronal gamma-band synchronization as a fundamental process in cortical computation. Annu. Rev. Neurosci. 32, 209–224. 10.1146/annurev.neuro.051508.135603 19400723

[B27] Gibson J. R. Bartley A. F. Hays S. A. Huber K. M. (2008). Imbalance of neocortical excitation and inhibition and altered UP states reflect network hyperexcitability in the mouse model of fragile X syndrome. J. Neurophysiol. 100, 2615–2626. 10.1152/jn.90752.2008 18784272 PMC2585391

[B28] Gonzalez-Burgos G. Cho R. Y. Lewis D. A. (2015). Alterations in cortical network oscillations and parvalbumin neurons in schizophrenia. Biol. Psychiatry 77, 1031–1040. 10.1016/j.biopsych.2015.03.010 25863358 PMC4444373

[B29] Gray L. J. Dean B. Kronsbein H. C. Robinson P. J. Scarr E. (2010). Region and diagnosis-specific changes in synaptic proteins in schizophrenia and bipolar I disorder. Psychiatry Res. 178, 374–380. 10.1016/j.psychres.2008.07.012 20488553

[B30] Halabisky B. Shen F. Huguenard J. R. Prince D. A. (2006). Electrophysiological classification of somatostatin-positive interneurons in mouse sensorimotor cortex. J. Neurophysiol. 96, 834–845. 10.1152/jn.01079.2005 16707715

[B31] Hata K. Polo-Parada L. Landmesser L. T. (2007). Selective targeting of different neural cell adhesion molecule isoforms during motoneuron myotube synapse formation in culture and the switch from an immature to mature form of synaptic vesicle cycling. J. Neurosci. 27, 14481–14493. 10.1523/JNEUROSCI.3847-07.2007 18160656 PMC6673458

[B32] Helmstaedter M. Sakmann B. Feldmeyer D. (2009). L2/3 interneuron groups defined by multiparameter analysis of axonal projection, dendritic geometry, and electrical excitability. Cereb. Cortex 19, 951–962. 10.1093/cercor/bhn130 18802122

[B33] Hunter P. R. Nikolaou N. Odermatt B. Williams P. R. Drescher U. Meyer M. P. (2011). Localization of Cadm2a and Cadm3 proteins during development of the zebrafish nervous system. J. Comp. Neurol. 519, 2252–2270. 10.1002/cne.22627 21456004

[B34] Ikeda M. Tomita Y. Mouri A. Koga M. Okochi T. Yoshimura R.. (2010). Identification of novel candidate genes for treatment response to risperidone and susceptibility for schizophrenia: integrated analysis among pharmacogenomics, mouse expression, and genetic case-control association approaches. Biol. Psychiatry 67, 263–269. 10.1016/j.biopsych.2009.08.030 19850283

[B35] Jiang X. Shen S. Cadwell C. R. Berens P. Sinz F. Ecker A. S.. (2015). Principles of connectivity among morphologically defined cell types in adult neocortex. Science 350:aac9462. 10.1126/science.aac9462 26612957 PMC4809866

[B36] Jurgenson M. Aonurm-Helm A. Zharkovsky A. (2012). Partial reduction in neural cell adhesion molecule (NCAM) in heterozygous mice induces depression-related behaviour without cognitive impairment. Brain Res. 1447, 106–118. 10.1016/j.brainres.2012.01.056 22361116

[B37] Kato H. K. Gillet S. N. Isaacson J. S. (2015). Flexible sensory representations in auditory cortex driven by behavioral relevance. Neuron 88, 1027–1039. 10.1016/j.neuron.2015.10.024 26586181 PMC4670799

[B38] Kätzel D. Zemelman B. V. Buetfering C. Wölfel M. Miesenböck G. (2011). The columnar and laminar organization of inhibitory connections to neocortical excitatory cells. Nat. Neurosci. 14, 100–107. 10.1038/nn.2687 21076426 PMC3011044

[B39] Kim J. Lee S. Tsuda S. Zhang X. Asrican B. Gloss B.. (2014). Optogenetic mapping of cerebellar inhibitory circuitry reveals spatially biased coordination of interneurons via electrical synapses. Cell Rep. 7, 1601–1613. 10.1016/j.celrep.2014.04.047 24857665 PMC4107211

[B40] Klausberger T. Márton L. F. Baude A. Roberts J. D. Magill P. J. Somogyi P. (2004). Spike timing of dendrite-targeting bistratified cells during hippocampal network oscillations *in vivo*. Nat. Neurosci. 7, 41–47. 10.1038/nn1159 14634650

[B41] Kochlamazashvili G. Senkov O. Grebenyuk S. Robinson C. Xiao M. F. Stummeyer K.. (2010). Neural cell adhesion molecule-associated polysialic acid regulates synaptic plasticity and learning by restraining the signaling through GluN2B-containing NMDA receptors. J. Neurosci. 30, 4171–4183. 10.1523/JNEUROSCI.5806-09.2010 20237287 PMC5390116

[B42] Kratz M. B. Manis P. B. (2015). Spatial organization of excitatory synaptic inputs to layer 4 neurons in mouse primary auditory cortex. Front. Neural Circuits 9:17. 10.3389/fncir.2015.00017 25972787 PMC4413692

[B43] Kuki T. Fujihara K. Miwa H. Tamamaki N. Yanagawa Y. Mushiake H. (2015). Contribution of parvalbumin and somatostatin-expressing GABAergic neurons to slow oscillations and the balance in beta-gamma oscillations across cortical layers. Front. Neural Circuits 9:6. 10.3389/fncir.2015.00006 25691859 PMC4315041

[B44] Lambo M. E. Turrigiano G. G. (2013). Synaptic and intrinsic homeostatic mechanisms cooperate to increase L2/3 pyramidal neuron excitability during a late phase of critical period plasticity. J. Neurosci. 33, 8810–8819. 10.1523/JNEUROSCI.4502-12.2013 23678123 PMC3700430

[B45] Le Pichon C. E. Firestein S. (2008). Expression and localization of the prion protein PrP^C^ in the olfactory system of the mouse. J. Comp. Neurol. 508, 487–499. 10.1002/cne.21698 18338400

[B46] Lewis D. A. Curley A. A. Glausier J. R. Volk D. W. (2012). Cortical parvalbumin interneurons and cognitive dysfunction in schizophrenia. Trends Neurosci. 35, 57–67. 10.1016/j.tins.2011.10.004 22154068 PMC3253230

[B47] Liu Z. Martin L. J. (2006). The adult neural stem and progenitor cell niche is altered in amyotrophic lateral sclerosis mouse brain. J. Comp. Neurol. 497, 468–488. 10.1002/cne.21012 16736475

[B48] Maness P. F. Schachner M. (2007). Neural recognition molecules of the immunoglobulin superfamily: signaling transducers of axon guidance and neuronal migration. Nat. Neurosci. 10, 19–26. 10.1038/nn1827 17189949

[B49] Mullins C. Fishell G. Tsien R. W. (2016). Unifying views of autism spectrum disorders: a consideration of autoregulatory feedback loops. Neuron 89, 1131–1156. 10.1016/j.neuron.2016.02.017 26985722 PMC5757244

[B50] Nelson S. B. Valakh V. (2015). Excitatory/inhibitory balance and circuit homeostasis in autism spectrum disorders. Neuron 87, 684–698. 10.1016/j.neuron.2015.07.033 26291155 PMC4567857

[B51] Packer A. M. Yuste R. (2011). Dense, unspecific connectivity of neocortical parvalbumin-positive interneurons: a canonical microcircuit for inhibition? J. Neurosci. 31, 13260–13271. 10.1523/JNEUROSCI.3131-11.2011 21917809 PMC3178964

[B52] Petreanu L. Huber D. Sobczyk A. Svoboda K. (2007). Channelrhodopsin-2-assisted circuit mapping of long-range callosal projections. Nat. Neurosci. 10, 663–668. 10.1038/nn1891 17435752

[B53] Petreanu L. Mao T. Sternson S. M. Svoboda K. (2009). The subcellular organization of neocortical excitatory connections. Nature 457, 1142–1145. 10.1038/nature07709 19151697 PMC2745650

[B54] Pillai-Nair N. Panicker A. K. Rodriguiz R. M. Gilmore K. L. Demyanenko G. P. Huang J. Z.. (2005). Neural cell adhesion molecule-secreting transgenic mice display abnormalities in GABAergic interneurons and alterations in behavior. J. Neurosci. 25, 4659–4671. 10.1523/JNEUROSCI.0565-05.2005 15872114 PMC6725026

[B55] Rao D. Basura G. J. Roche J. Daniels S. Mancilla J. G. Manis P. B. (2010). Hearing loss alters serotonergic modulation of intrinsic excitability in auditory cortex. J. Neurophysiol. 104, 2693–2703. 10.1152/jn.01092.2009 20884760 PMC2997032

[B56] Richardson M. J. Silberberg G. (2008). Measurement and analysis of postsynaptic potentials using a novel voltage-deconvolution method. J. Neurophysiol. 99, 1020–1031. 10.1152/jn.00942.2007 18046003

[B57] Rudy B. Fishell G. Lee S. Hjerling-Leffler J. (2011). Three groups of interneurons account for nearly 100% of neocortical GABAergic neurons. Dev. Neurobiol. 71, 45–61. 10.1002/dneu.20853 21154909 PMC3556905

[B58] Senkov O. Sun M. Weinhold B. Gerardy-Schahn R. Schachner M. Dityatev A. (2006). Polysialylated neural cell adhesion molecule is involved in induction of long-term potentiation and memory acquisition and consolidation in a fear-conditioning paradigm. J. Neurosci. 26, 10888–109898. 10.1523/JNEUROSCI.0878-06.2006 17050727 PMC6674738

[B59] Sohal V. S. Zhang F. Yizhar O. Deisseroth K. (2009). Parvalbumin neurons and gamma rhythms enhance cortical circuit performance. Nature 459, 698–702. 10.1038/nature07991 19396159 PMC3969859

[B60] Stensrud M. J. Chaudhry F. A. Leergaard T. B. Bjaalie J. G. Gundersen V. (2013). Vesicular glutamate transporter-3 in the rodent brain: vesicular colocalization with vesicular gamma-aminobutyric acid transporter. J. Comp. Neurol. 521, 3042–3056. 10.1002/cne.23331 23633129

[B61] Stork O. Welzl H. Wotjak C. T. Hoyer D. Delling M. Cremer H.. (1999). Anxiety and increased 5-HT1_A_ receptor response in NCAM null mutant mice. J. Neurobiol. 40, 343–355. 10.1002/(SICI)1097-4695(19990905)40:3<343::AID-NEU6>3.0.CO;2-S 10440734

[B62] Tikidji-Hamburyan R. A. Martinez J. J. White J. A. Canavier C. C. (2015). Resonant interneurons can increase robustness of gamma oscillations. J. Neurosci. 35, 15682–15695. 10.1523/JNEUROSCI.2601-15.2015 26609160 PMC4659828

[B63] Toader O. Forte N. Orlando M. Ferrea E. Raimondi A. Baldelli P.. (2013). Dentate gyrus network dysfunctions precede the symptomatic phase in a genetic mouse model of seizures. Front. Cell. Neurosci. 7:138. 10.3389/fncel.2013.00138 24009558 PMC3757301

[B64] Varea E. Guirado R. Gilabert-Juan J. Marti U. Castillo-Gomez E. Blasco-Ibanez J. M.. (2012). Expression of PSA-NCAM and synaptic proteins in the amygdala of psychiatric disorder patients. J. Psychiatr. Res. 46, 189–197. 10.1016/j.jpsychires.2011.10.011 22099865

[B65] Volman V. Behrens M. M. Sejnowski T. J. (2011). Downregulation of parvalbumin at cortical GABA synapses reduces network gamma oscillatory activity. J. Neurosci. 31, 18137–18148. 10.1523/JNEUROSCI.3041-11.2011 22159125 PMC3257321

[B66] Wang H. Peca J. Matsuzaki M. Matsuzaki K. Noguchi J. Qiu L.. (2007). High-speed mapping of synaptic connectivity using photostimulation in Channelrhodopsin-2 transgenic mice. Proc. Natl. Acad. Sci. U S A 104, 8143–8148. 10.1073/pnas.0700384104 17483470 PMC1876585

[B67] Wang T. W. Stromberg G. P. Whitney J. T. Brower N. W. Klymkowsky M. W. Parent J. M. (2006). Sox3 expression identifies neural progenitors in persistent neonatal and adult mouse forebrain germinative zones. J. Comp. Neurol. 497, 88–100. 10.1002/cne.20984 16680766

[B68] Wilson G. M. Flibotte S. Chopra V. Melnyk B. L. Honer W. G. Holt R. A. (2006). DNA copy-number analysis in bipolar disorder and schizophrenia reveals aberrations in genes involved in glutamate signaling. Hum. Mol. Genet. 15, 743–749. 10.1093/hmg/ddi489 16434481

[B70] Xu X. Roby K. D. Callaway E. M. (2010). Immunochemical characterization of inhibitory mouse cortical neurons: three chemically distinct classes of inhibitory cells. J. Comp. Neurol. 518, 389–404. 10.1002/cne.22229 19950390 PMC2804902

[B69] Xu Q. Tam M. Anderson S. A. (2008). Fate mapping Nkx2.1-lineage cells in the mouse telencephalon. J. Comp. Neurol. 506, 16–29. 10.1002/cne.21529 17990269

[B71] Yizhar O. Fenno L. E. Davidson T. J. Mogri M. Deisseroth K. (2011). Optogenetics in neural systems. Neuron 71, 9–34. 10.1016/j.neuron.2011.06.004 21745635

[B72] Zhao S. Ting J. T. Atallah H. E. Qiu L. Tan J. Gloss B.. (2011). Cell type-specific channelrhodopsin-2 transgenic mice for optogenetic dissection of neural circuitry function. Nat. Methods 8, 745–752. 10.1038/nmeth.1668 21985008 PMC3191888

